# PP2A regulates shade avoidance response by dephosphorylating phytochrome‐interacting factor 7 in Arabidopsis

**DOI:** 10.1111/tpj.71073

**Published:** 2026-08-02

**Authors:** Xingbo Cai, Oihik Mitra, Amariah Gustamante, Wenqiang Tang, Yu Sun, Enamul Huq

**Affiliations:** ^1^ Department of Molecular Biosciences and the Institute for Cellular and Molecular Biology The University of Texas at Austin Austin Texas 78712 USA; ^2^ Hebei Key Laboratory of Molecular and Cellular Biology, College of Life Sciences Hebei Normal University Shijiazhuang Hebei 050024 China

**Keywords:** protein phosphatase, PP2A, phytochrome‐interacting factors (PIFs), phytochromes, shade‐avoidance syndrome (SAS), Arabidopsis

## Abstract

Shade avoidance is a key adaptive growth response that enables plants to compete for light under dense vegetation. In *Arabidopsis thaliana*, the transcription factor PHYTOCHROME‐INTERACTING FACTOR 7 (PIF7) is dephosphorylated under shade and accumulates into the nucleus to promote shade‐induced elongation growth through activation of auxin biosynthesis/signaling genes, yet the molecular mechanism underlying PIF7 dephosphorylation under shade conditions has remained unclear. Using yeast two‐hybrid screening and extensive biochemical, genetic, and photobiological analyses, we identified protein phosphatase 2A (PP2A) as a direct regulator of PIF7 activity. We show that four PP2A regulatory subunits, B′α, B′β, B″α, and B″β, associate with PIF7 both *in vitro* and *in vivo*. Genetic analyses demonstrate that loss of PP2A B′α, B′β, B″α, and B″β function reduces hypocotyl elongation under shade, whereas overexpression of these subunits enhances shade‐induced growth. Epistasis analyses further reveal that PP2A acts in the same genetic pathway as PIF7 to regulate hypocotyl elongation during shade avoidance. Consistently, expression of PIF7 target genes is attenuated in *pp2a* mutants under shade conditions, and immunopurified PP2A complexes efficiently dephosphorylate PIF7 *in vitro*. Together, our findings establish a direct biochemical role for PP2A in PIF7 activation and reveal a new role for PP2A in regulating shade responses in plants.

## Significance Statement

The shade‐avoidance response allows plants to better access sunlight to compete against surrounding neighbors. In Arabidopsis, PIF7 can be dephosphorylated under shade and accumulates into the nucleus to promote auxin biosynthetic and signaling genes to promote stem elongation. However, how PIF7 is dephosphorylated under shade condition and which phosphatases are involved in this process are not known. Here we show that four PP2A B subunits dephosphorylate PIF7 to promote shade‐avoidance responses in Arabidopsis.

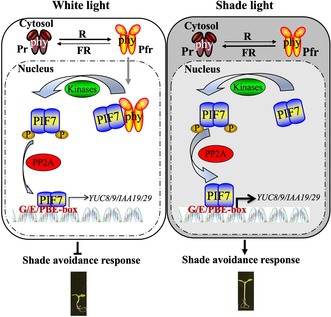

## INTRODUCTION

As sessile organisms, plants must continuously adjust their growth and development to cope with fluctuating environmental conditions. In shade‐avoiding species such as *Arabidopsis thaliana*, exposure to vegetative shade, characterized by a low red to far‐red (R:FR) light ratio, triggers a suite of morphological changes collectively known as the shade‐avoidance syndrome (SAS). These include elongation of the hypocotyl, stem, and petioles, accompanied by reduced leaf expansion and increased upward leaf mobility (hyponasty), allowing plants to outcompete neighboring vegetation by improving access to sunlight (Casal, 2013; Gautrat et al., 2025).

A central regulator of shade‐avoidance signaling is the red/far‐red photoreceptor phytochrome B (phyB) (Reed et al., 1993). Under sunlight (R:FR > 1), phyB adopts its active Pfr form and accumulates in the nucleus within discrete photobodies (Van Buskirk et al., 2011). Active phyB interacts with PHYTOCHROME‐INTERACTING FACTORS (PIFs), a family of basic helix–loop–helix (bHLH) transcription factors, promoting their phosphorylation and subsequent degradation, with the notable exception of PIF7 (Cheng et al., 2021). This phyB‐mediated repression of PIF activity prevents excessive elongation growth. By contrast, under shade conditions (R:FR < 1), phyB is photoconverted to the inactive Pr form, leading to the accumulation and activation of PIFs that promote hypocotyl elongation (Legris et al., 2019). Among the PIF family members, PIF4, PIF5, and PIF7 positively regulate shade‐induced growth; however, genetic analyses identify PIF7 as a major regulator of this response, as *pif7* mutants display a strongly impaired shade‐avoidance phenotype (Gautrat et al., 2025; Li et al., 2012).

Unlike other PIFs, PIF7 undergoes a distinctive mode of post‐translational regulation. Under white light conditions, PIF7 exists in both phosphorylated and unphosphorylated forms (Li et al., 2012). Phosphorylated PIF7 associates with 14‐3‐3 proteins and is retained in the cytoplasm (Huang et al., 2018), whereas the unphosphorylated form accumulates in the nucleus. Under white light, nuclear PIF7 colocalizes with phyB in discrete photobodies, where the formation of PIF7–phyB condensates suppresses the DNA‐binding activity of PIF7 (Xie et al., 2023). Upon exposure to shade, PIF7 undergoes rapid dephosphorylation and nuclear accumulation, enabling it to bind the promoters of auxin biosynthesis and signaling genes, such as *YUCCA8* and *IAA19*, thereby promoting hypocotyl and petiole elongation (Huang et al., 2018).

Recent studies further reveal that PIF7 integrates signals from multiple photoreceptor pathways. PIF7 interacts with phyA and the FHY1/FHL shuttling machinery to form photobody‐localized complexes that attenuate phyA nuclear import and signaling under far‐red–enriched shade conditions (Zhou et al., 2025). In addition, PIF7 protein abundance is regulated by the ubiquitin‐specific proteases UBP12 and UBP13 (Zhou et al., 2021). PIF7 also activates the expression of a long non‐coding RNA (lncRNA), called PHYA UTR Antisense RNA (PUAR), which blocks the binding of PIF7 to the *PHYA* promoter, hindering feedback regulation of PIF7‐phyA circuitry and promotion of the hypocotyl elongation response under low R:FR light conditions (Zhu et al., 2023). Together, these findings highlight PIF7 as a multifaceted regulator positioned at the intersection of multiple light‐signaling pathways. Despite these advances, the molecular mechanism underlying shade‐induced PIF7 dephosphorylation remains unknown.

Protein phosphatases have been shown to play crucial roles in cellular signaling pathways (Farkas et al., 2007; Luan, 2003). They are broadly classified into Type 1 (PP1) and Type 2 (PP2) phosphatases. PP2 can be further classified into PP2A, PP2B and PP2C phosphatases (Luan, 2003). Among these, PP2A is composed of three subunits: A (scaffold), B (regulatory) and C (catalytic) subunits. Arabidopsis genome encodes 3 isoforms of A subunit, 5 isoforms of C subunit and 17 isoforms of B subunits (Farkas et al., 2007). The B subunit determines substrate specificity and subcellular localization. Previously, several phosphatases have been shown to regulate PIF phosphorylation status. TYPE‐ONE PROTEIN PHOSPHATASE4 (TOPP4) is a catalytic subunit of PP1 that controls the phosphorylation status of PIF5 (Yue et al., 2016). FyPP1 and FyPP2 are the catalytic subunits of PP6 that have been shown to dephosphorylate PIF3/4 and regulate their abundance (Yu et al., 2019). Recently, we have shown that Arabidopsis PP2A regulatory subunits B′α, B′β, B″α, and B″β interacted with and dephosphorylated PIF3 to regulate red light signaling (Cai et al., 2024). However, the phosphatase(s) that control PIF7 phosphorylation status have not been identified yet.

Here, we demonstrate that PP2A directly dephosphorylates PIF7 to promote shade‐induced hypocotyl elongation in Arabidopsis. We show that the PP2A regulatory subunits B′α, B′β, B″α, and B″β interact with PIF7 both *in vitro* and *in vivo*. Phenotypic analyses of single and higher‐order mutants reveal that these PP2A subunits function in the same genetic pathway as PIF7 to regulate hypocotyl elongation under shade conditions. Consistently, the presence of these regulatory subunits enhances shade‐induced PIF7 dephosphorylation, and immunopurified PP2A complexes efficiently dephosphorylate PIF7 *in vitro*. Together, our findings uncover a PP2A‐mediated mechanism that activates PIF7 during shade avoidance and provide new insight into the post‐translational regulation of light‐controlled growth response in plants.

## RESULTS

### PP2A promotes shade light‐induced hypocotyl elongation

**Figure 1:**
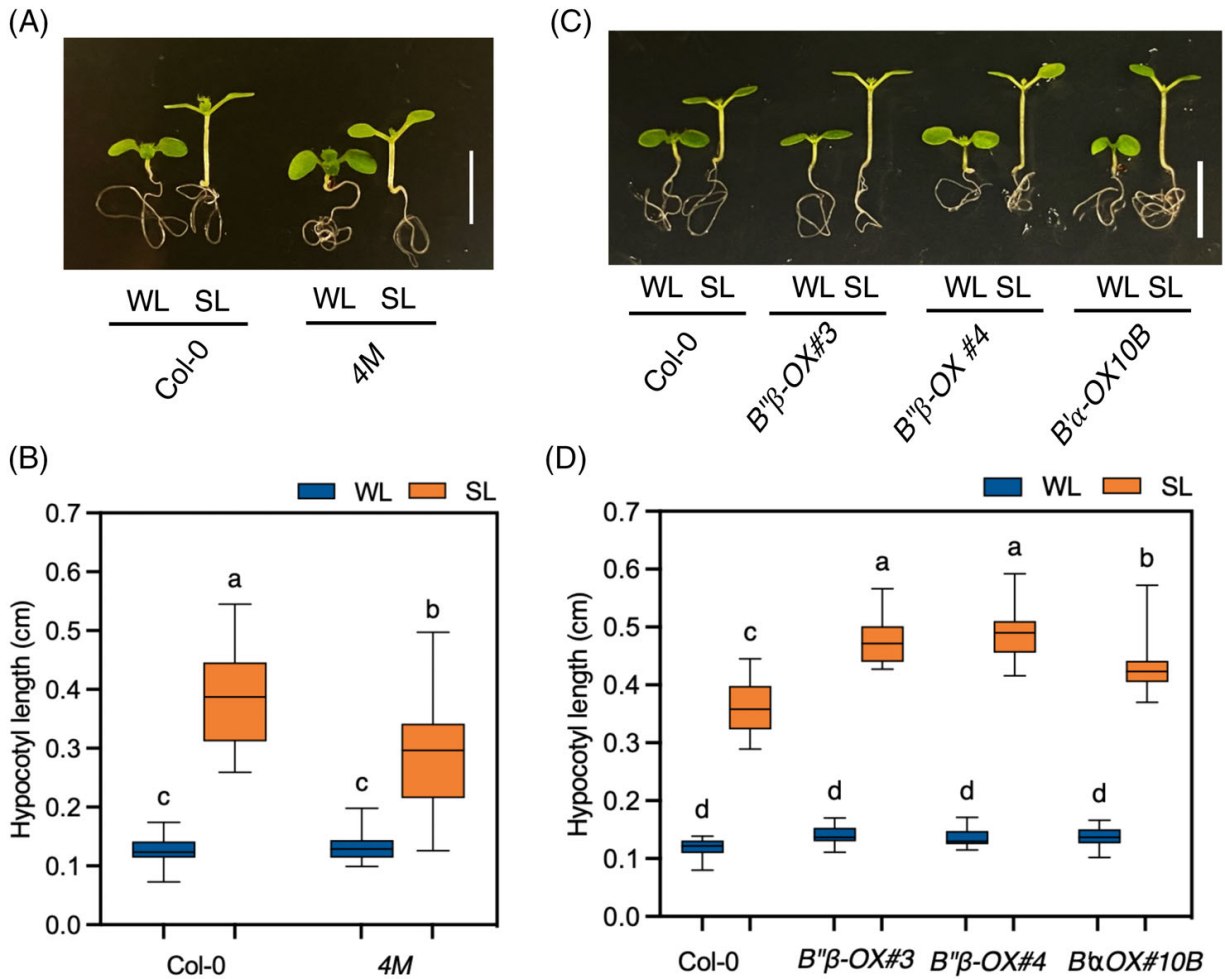
PP2A promotes shade‐induced hypocotyl elongation: Seedlings were grown under white light for 3 days to allow germination then either kept in the white light (22°C) or continuous shade light conditions for 6 days. (A) Photographs of seedlings showing hypocotyl length: The *pp2a b″αβ/b′αβ* quadruple mutant (*4M*) hypocotyl length is shown compared to wild type (Col‐0) under white light and shade light condition. (B) The boxplots exhibit the hypocotyl lengths of seedlings shown in (A). (C) Photographs of seedlings showing hypocotyl length: Independent overexpression lines of PP2A B subunits (*B″β‐OX#3, B″β‐OX#4, B′α‐OX10B*) hypocotyl length is shown compared to wild type (Col‐0) under white light and shade light condition. (D) The boxplots exhibit the hypocotyl lengths of seedlings shown in (C). Three biological replicates of >20 seedlings were used for measurements. The error bars represent SEM. A one‐way anova followed by Tukey's multiple comparison test was performed. Statistically significant differences are indicated by different lowercase letters (*P* < 0.05). SL, shade light; WL, white light.

Previously, we showed that PP2A inhibits photomorphogenesis by dephosphorylating PIF3 in Arabidopsis (Cai et al., 2024). PP2A B subunits have been reported to localize in both the cytosol and nucleus (Cai et al., 2024). Because PIF7 is also present in both compartments, we hypothesized that PP2A might control the phosphorylation status of PIF7 and thereby regulate shade‐avoidance responses. To test this hypothesis, we analyzed hypocotyl elongation in PP2A mutants and overexpression lines under both shade and white light conditions. Our results showed that the *pp2a b″α‐pp2ab″β* (hereafter referred to as *b″αβ*), *pp2ab′α‐pp2ab′β* (hereafter referred to as *b′αβ*) double mutants and *pp2ab″αβ/b′αβ* quadruple mutant (hereafter referred to as *4M*) displayed significantly shorter hypocotyls under shade conditions compared with the wild type (Col‐0), whereas hypocotyl lengths were similar between the mutants and the wild type under white light conditions (Figure 1A,B; Figure S1). In contrast, overexpression of PP2A B subunits resulted in longer hypocotyls under shade conditions compared to the wild type (Figure 1C,D; Figure S2). Because the PP2A functions as a heterotrimeric enzyme composed of A, B and C subunits, we next examined hypocotyl phenotypes in PP2A A subunit overexpression lines under both shade and white light conditions. PP2A A subunit overexpression lines also exhibited longer hypocotyls under shade conditions compared with the wild type, suggesting PP2A A subunit contributes to the regulation of shade‐induced hypocotyl elongation. Notably, both PP2A A and B overexpression lines showed slightly longer hypocotyls under white light conditions (Figure 1; Figure S2), suggesting that PP2A also functions under white light conditions. Together, these results demonstrate that PP2A positively regulates shade light‐induced hypocotyl elongation in Arabidopsis.

### PP2A B′α, B′β, B″α and B″β interact with PIF7

**Figure 2:**
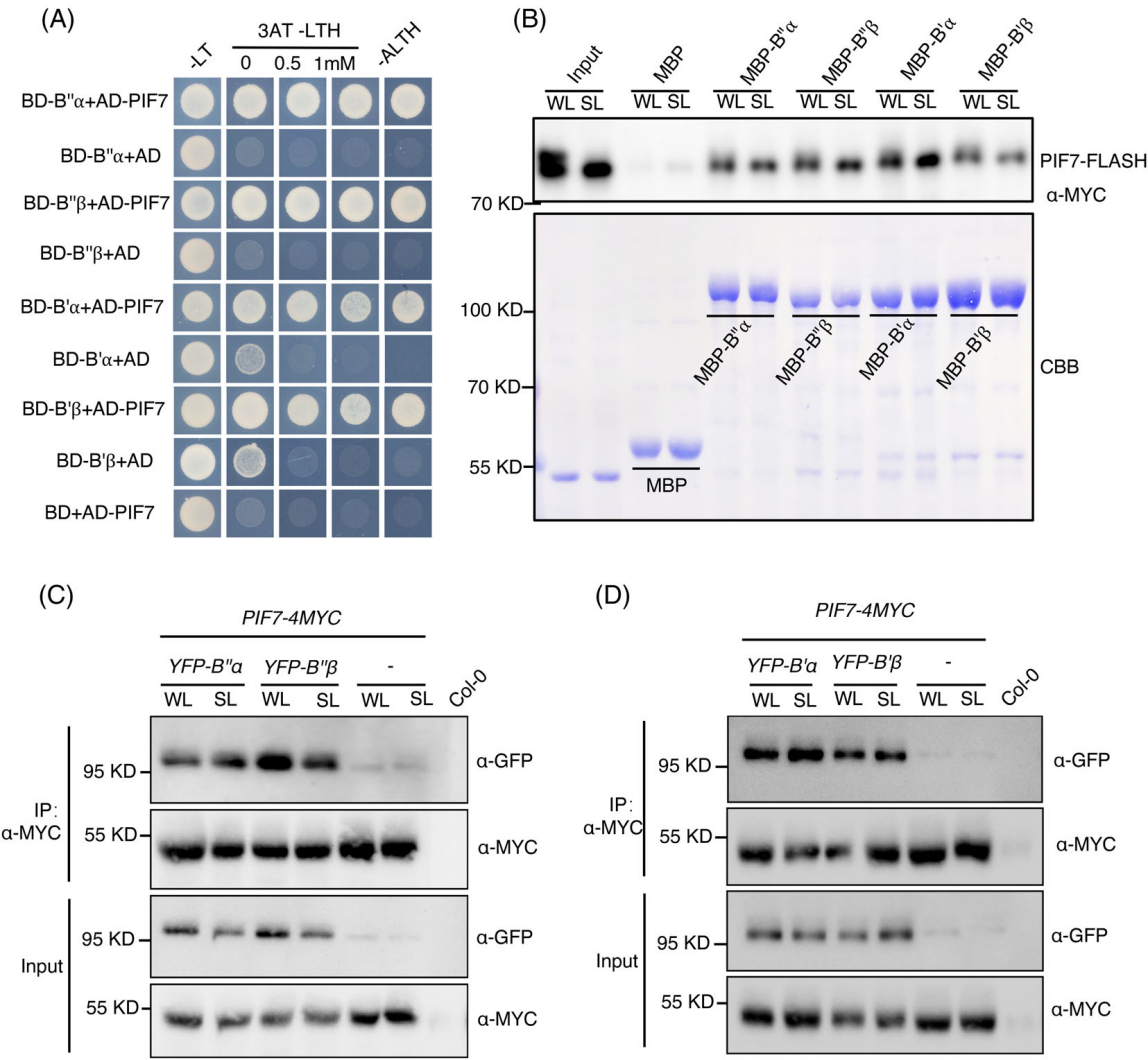
PIF7 interacts with PP2A B″α, B″β, B′α and B′β *in vitro* and *in vivo*. (A) Yeast two‐hybrid (Y2H) assays showing the interaction between full‐length PIF7 and PP2A B″α, B″β, B′α and B′β subunits. The B″α, B″β, B′α and B′β‐GAL4 DNA‐binding domain fusions (BD‐B″α, BD‐B″β, BD‐B′α and BD‐B′β) were co‐expressed with the GAL4 activation domain (AD) fused to full‐length PIF7 or with AD alone as a negative control. Yeast cells were grown on selective media lacking histidine and supplemented with increasing concentrations of the histidine biosynthesis inhibitor 3‐amino‐1,2,4‐triazole (3‐AT). –ALTH, medium lacking Ade, Leu, Trp, and His amino acids; –LT, medium lacking Leu and Trp amino acids; –LTH, medium lacking Leu, Trp, and His amino acids. (B) Semi‐*in vivo* pull‐down assays showing the interaction between PIF7‐FLASH and MBP‐B″α, MBP‐B″β, MBP‐B′α or MBP‐B′β. MBP‐tagged proteins were incubated with extracts from 5 day‐old white light grown seedlings of the *35S:PIF7‐FLASH* transgenic line (either white or 1 h shade light treated), followed by pull‐down using MBP beads. PIF7‐FLASH signals were detected on the membrane using an α‐MYC antibody. MBP alone served as a negative control. Gel was stained with CBB to show the presence of MBP‐tagged proteins. Inputs from white light and shade light‐treated extracts were used as positive controls. CBB, Coomassie Brilliant Blue. (C, D) *In vivo* co‐immunoprecipitation (co‐IP) assays showing that PIF7‐MYC interaction with B″α‐GFP and B″β‐GFP (C) and PIF7‐MYC interaction with B′α‐GFP and B′β‐GFP (D) under both white light and shade light conditions. Four‐day‐old white light‐grown seedlings of *35S:YFP‐B″α/PIF7‐MYC, 35S:YFP‐B″β/PIF7‐MYC, 35S:YFP‐B′α/PIF7‐MYC, 35S:YFP‐B′β/PIF7‐MYC, PIF7‐MYC,* and Col‐0 were used. *PIF7‐MYC* and Col‐0 served as negative controls. One batch was maintained in white light, and another was exposed to shade light treatment for 20 min. All seedlings were treated with 100 μm Bortezomib for 4 h in darkness. α‐MYC antibody was used for immunoprecipitation of PIF7‐MYC and α‐GFP antibody was used to detect YFP‐B″α and YFP‐B″β, YFP‐B′α and YFP‐B′β. CBB, Coomassie Brilliant Blue stain; SL, Shade light; WL, White light.

Because both PIFs and PP2A regulate shade light‐induced hypocotyl elongation, we asked whether PP2A interacts with any PIFs to regulate the shade response. Among the PIF family members, PIF4, PIF5, and PIF7 contribute to shade‐induced growth, with PIF7 acting as the major regulator. Given that PP2A regulatory subunits B′α, B′β, B″α and B″β interact with PIF3 and PIF4 *in vitro*, we investigated whether these PP2A subunits also interact with PIF7. Using yeast two‐hybrid (Y2H) and semi‐*in vivo* pull‐down assays, we found that PP2A B′α, B′β, B″α and B″β exhibit strong interactions with PIF7 (Figure 2A,B), whereas no significant interaction was found with RCN1 and A3 subunits in yeast two‐hybrid assays (Figure S3). To further validate these interactions *in vivo*, we overexpressed each of the four PP2A B subunits in the *35S:PIF7‐4MYC* background, which showed an exaggerated hypocotyl phenotype under shade light (Figure S4A,B). These lines were used to perform co‐immunoprecipitation (Co‐IP) assays. PIF7 and PP2A B′α, B′β, B″α and B″β show strong interactions *in vivo* (Figure 2C,D; Figure S5). Together, these results indicate that PP2A B′α, B′β, B″α and B″β subunits interact with PIF7 both *in vivo* and *in vitro*, supporting a role for PP2A in regulating shade‐avoidance responses through direct association with PIF7.

### PP2A B′α, B′β, B″α, B″β and PIF7 function in the same genetic pathway to regulate shade response

**Figure 3:**
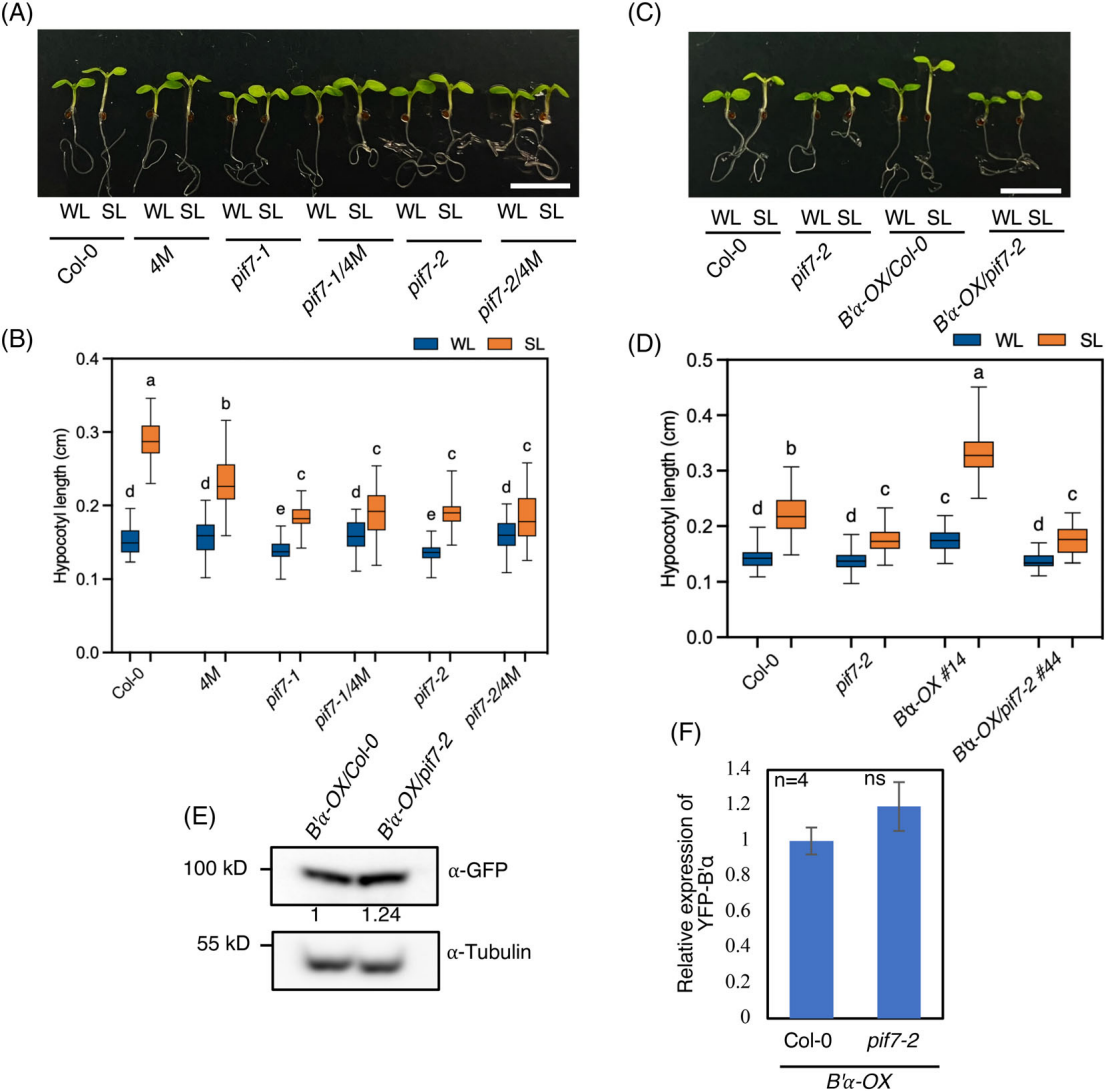
PP2A B′α, B′β, B″α, B″β and PIF7 function in the same genetic pathway to regulate shade response. Seedlings were grown under white light for 3 days to allow germination, then either kept in white light (22°C) or transferred to continuous shade light conditions. Seedlings were grown under these conditions for 6 days. (A) Photographs of seedlings showing hypocotyl length. The *pp2a b″αβ/b′αβ* quadruple mutant (*4M*), *pif7* (*pif7‐1*, *pif7‐2*), and *pif7/4M* quintuple mutants (*pif7‐1/4M*, *pif7 2/4M*) hypocotyls are shown compared to wild type (Col‐0) under white light and shade light conditions. (B) Boxplots showing quantification of hypocotyl lengths for the seedlings shown in (A). (C) Photographs of seedlings showing hypocotyl length. The PP2A B′α overexpression line (*B′α‐OX*/Col‐0) and its cross with *pif7‐2* (*B′α‐OX/pif7‐2*) are compared to Col‐0 and *pif7‐2* under white and shade light conditions. (D) Boxplots show quantification of hypocotyl lengths for the seedlings shown in (C). (E) Immunoblot analysis shows PP2A B′α protein accumulation in *B′α‐OX*/Col‐0 and *B′α‐OX*/*pif7‐2* seedlings. α Tubulin antibody was used to show as a loading control. (F) Bar graph shows quantification of data shown in (E). Three biological replicates of >20 seedlings were used for measurements. Error bars represent SEM. Statistical significance was determined by one‐way anova followed by Tukey's multiple comparison test, and different lowercase letters indicate significant differences (*P* < 0.05). Error bars in (F) represent SEM (*n* = 4). *P* < 0.01, based on Student's *t*‐test. SL, shade light; WL, white light.

Because PP2A interacts with PIF7 and positively regulates shade‐induced hypocotyl elongation, we hypothesized that PP2A and PIF7 might act in the same genetic pathway to regulate the shade responses. To test this hypothesis, we generated the *4M/pif7* quintuple mutant by introducing two independent *pif7* alleles into *4M* quadruple mutant background and examined hypocotyl elongation under white light and shade conditions. Under shade conditions, the *4M* mutant exhibited shorter hypocotyls than the wild type but longer hypocotyls than the *pif7* single mutant (Figure 3A,B). In contrast, both *4M/pif7* quintuple mutants displayed hypocotyl lengths comparable to those of the corresponding *pif7* single mutant (Figure 3A,B). Consistently, *b″αβ/pif7‐2* lines exhibited hypocotyl phenotypes largely indistinguishable from *pif7‐2* under shade conditions (Figure S6A,B). These results indicate that *pif7* is epistatic to *pp2a*, placing PP2A upstream of PIF7 in the genetic pathway regulating shade‐induced hypocotyl elongation.

To further assess whether the elongated hypocotyl phenotype observed in PP2A overexpression lines depends on PIF7, we generated the *B′α‐OX/pif7* line. As expected, *B′α‐OX/Col‐0* seedlings displayed longer hypocotyls than the wild type under shade conditions (Figure 3C,D). However, *B′α‐OX/pif7‐2* exhibited hypocotyl lengths similar to those of the *pif7‐2* single mutant (Figure 3C,D), despite comparable accumulation of B′α protein in the *pif7‐2* and Col‐0 backgrounds (Figure 3E,F). Together, these findings demonstrate that PP2A promotes shade‐induced hypocotyl elongation in a PIF7‐dependent manner and that PP2A and PIF7 function in the same genetic pathway to regulate shade responses.

### PP2A regulates PIF7 phosphorylation status

**Figure 4:**
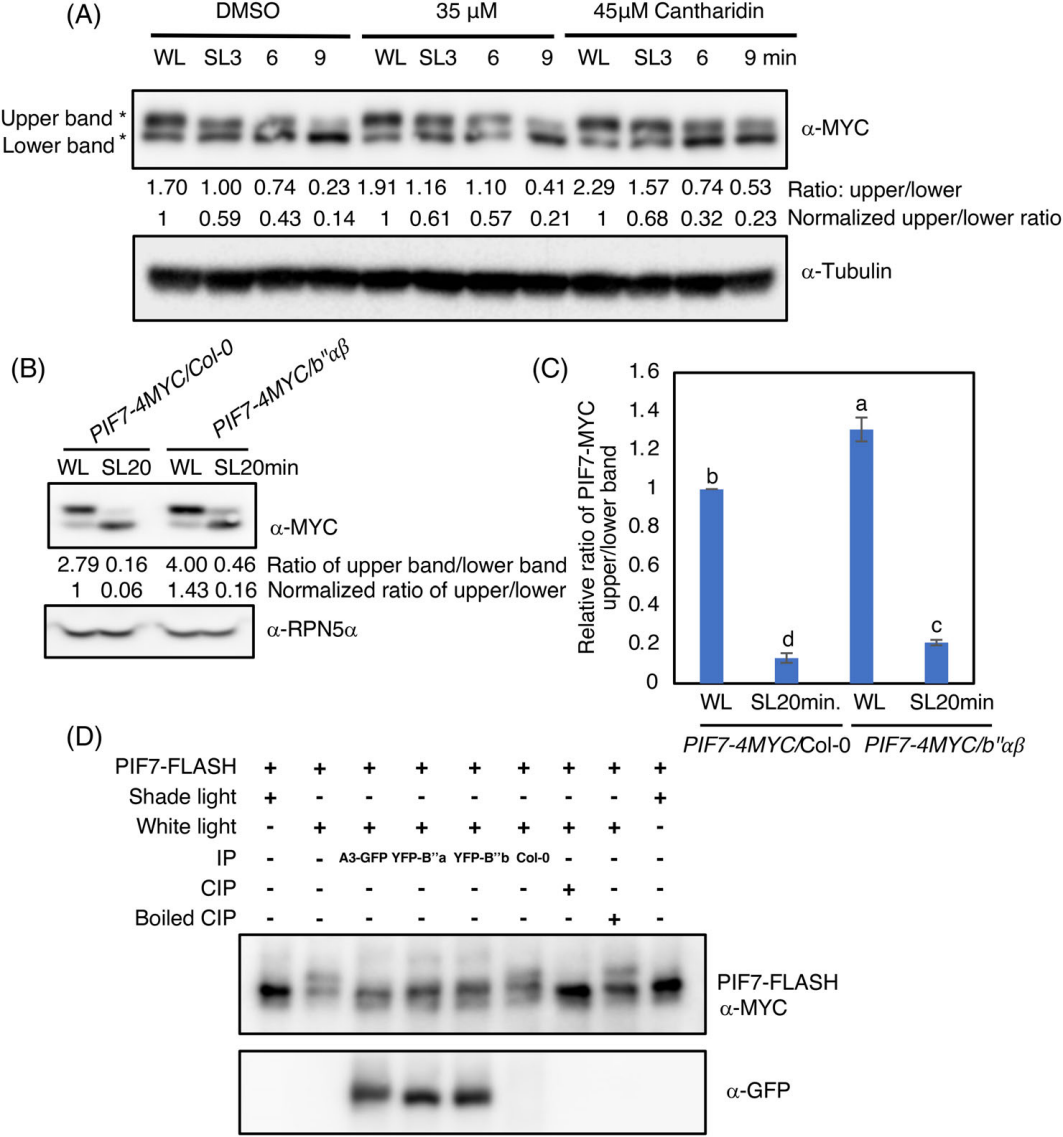
PP2A regulates PIF7 phosphorylation status and directly dephosphorylates PIF7 *in vitro*. (A) Immunoblots showing that the PP2A inhibitor Cantharidin affects PIF7‐FLASH dephosphorylation. Five‐day‐old white light‐grown *35S:PIF7 FLASH* seedlings were pre‐treated with 100 μm Bortezomib for 4 h to block protein degradation and subsequently treated with DMSO (control) or Cantharidin (35 μm or 45 μm) for 30 min before shade light treatment for the indicated durations. Upper and lower PIF7‐FLASH bands represent phosphorylated and unphosphorylated forms respectively, The upper/lower band ratios are shown below the α‐MYC blot along with the normalized ratio relative to the loading control (Tubulin). (B) Immunoblots showing the PIF7‐4MYC dephosphorylation pattern in Col‐0 and *pp2ab″αβ *(*b″αβ*) backgrounds. Five‐day‐old white light‐grown seedlings were pre‐treated with 100 μm Bortezomib and 100 μm MG132 for 4 h in darkness and then exposed to shade light for 20 min. PIF7‐4MYC was detected using α‐MYC antibody. Upper and lower PIF7‐4MYC bands represent phosphorylated and unphosphorylated forms, respectively. The upper/lower band ratios are shown below the α‐MYC blot along with the normalized ratio relative to the loading control (RPN5a). (C) Quantification of the relative PIF7‐4MYC upper/lower band ratios shown in (B). The ratio in Col‐0 was set as 1. Error bars represent SEM (*n* = 3). *P* < 0.01, based on one‐way anova followed by Tukey's multiple comparison test. (D) Dephosphorylation assay was performed using immunoprecipitated PP2A complexes and PIF7‐FLASH as substrate. PP2A proteins were immunoprecipitated from *35S:A3‐GFP*, *35S:YFP‐B″α*, and *35S:YFP‐B″β* overexpression lines and incubated with PIF7‐FLASH‐containing extracts from white light‐grown seedlings for 1 h at 30°C. CIP served as a positive control, while boiled CIP and Col‐0 IP products served as negative controls. α‐MYC antibody was used for detection. CIP, calf intestinal phosphatase; SL, shade light; WL, white light.

*35S:PIF7‐FLASH*, a tagged PIF7 line, has been shown to be phosphorylated under white light and rapidly dephosphorylated upon exposure to shade light (Huang et al., 2018; Li et al., 2012; Zhou et al., 2021). Therefore, we examined the dynamics of PIF7 phosphorylation and dephosphorylation under our experimental light conditions. As shown in Figure S7A, PIF7‐FLASH was dephosphorylated within 10 min of shade light exposure and became re‐phosphorylated following subsequent white light treatments of different durations. To determine whether PP2A affects PIF7‐FLASH dephosphorylation, we first evaluated the effect of the PP2A inhibitor cantharidin on PIF7‐FLASH dephosphorylation. Five‐day‐old white light‐grown seedlings of PIF7‐FLASH were pre‐treated with bortezomib to inhibit 26S proteasome‐mediated PIF7‐FLASH degradation, followed by treatment with cantharidin for 30 min before being transferred to shade for the indicated times. Under white light conditions, cantharidin treatment (35 μm or 45 μm) resulted in increased accumulation of the slower‐migrating phosphorylated bands compared to the DMSO control (Figure 4A), indicating that cantharidin inhibits PIF7‐FLASH dephosphorylation under white light conditions. Because bortezomib blocks the 26S proteasome‐mediated degradation pathway, phosphorylated PIF7‐FLASH was stabilized under these conditions. Following shade exposure, cantharidin treatment also delayed PIF7‐FLASH dephosphorylation relative to the DMSO treatment (Figure 4A). Consistently, cantharidin treatment reduced the long hypocotyl phenotype of PIF7‐FLASH compared to control under shade conditions (Figure S7B,C). Together, these results suggest that inhibition of PP2A activity partially suppresses PIF7‐FLASH dephosphorylation under both white and shade light conditions and impairs PIF7 function.

To genetically assess the role of PP2A in regulating PIF7 phosphorylation status, we generated *35S:PIF7‐4MYC* transgenic lines in multiple genetic backgrounds. To confirm the functionality of the construct, *35S:PIF7‐4MYC* was first introduced into the *pif7‐2* mutant background. Overexpression of *35S:PIF7‐4MYC* resulted in markedly elongated hypocotyls under shade conditions compared with the wild type (Figure S8A,B). In addition, shade light exposure led to a reduction in the phosphorylated form of PIF7‐4MYC and accumulation of the unphosphorylated PIF7‐4MYC form, closely resembling the PIF7‐FLASH band shift pattern observed after shade treatment (Figure S8C). These results indicate that PIF7‐4MYC is functionally active in plants. We next introduced *35S:PIF7‐4MYC* into the wild type and *b″αβ* backgrounds and selected lines with comparable PIF7‐4MYC expression levels in both backgrounds (Figure 4B; Figure S8D). Hypocotyl measurements under white and shade light conditions showed that *b″αβ* mutation significantly attenuated the elongated hypocotyl phenotype of *35S:PIF7‐4MYC/*Col‐0 under shade conditions (Figure S9A,B). Conversely, double overexpression lines expressing PIF7‐4MYC and all four YFP‐B subunits displayed longer hypocotyls compared to the PIF7‐4MYC line (Figure S4A,B). These findings support a role for PP2A in regulating PIF7 phosphorylation status and activity during shade‐induced growth responses.

To test whether PP2A affects PIF7‐4MYC dephosphorylation, we used the lines described above to examine PIF7‐4MYC dephosphorylation dynamics in the wild type and *b″αβ* backgrounds. Five‐day‐old white light‐grown *35S:PIF7‐4MYC*/Col‐0 and *35S:PIF7‐4MYC*/*b″αβ* seedlings were pre‐treated with bortezomib and MG132 for 4 h to inhibit PIF7‐4MYC degradation and subsequently exposed to shade light for 20 min to induce PIF7‐4MYC dephosphorylation. The ratio of upper (phosphorylated) to lower (unphosphorylated) PIF7‐4MYC bands was calculated as an indicator of phosphorylation status. As shown in Figure 4B,C, a higher proportion of phosphorylated PIF7‐4MYC accumulated in the *b″αβ* background under white light conditions compared with wild type background, indicating that PP2A contributes to PIF7‐4MYC dephosphorylation under white light conditions. This result is consistent with the cantharidin‐mediated accumulation of phosphorylated PIF7‐FLASH observed under white light relative to the DMSO control (Figure 4A). Following shade treatment, the level of phosphorylated PIF7‐4MYC remained significantly higher in *b″αβ* background than in the wild type, indicating PP2A is also involved in shade light‐induced PIF7‐4MYC dephosphorylation.

As shown in Figure S7A, unphosphorylated PIF7 can be re‐phosphorylated upon transfer back to white light. We therefore examined whether PP2A also affects the kinetics of PIF7‐4MYC re‐phosphorylation under white light conditions. Five‐day‐old white light‐grown PIF7‐4MYC/Col‐0 and PIF7‐4MYC/*b″αβ* seedlings were pre‐treated with bortezomib and MG132 for 4 h, exposed to shade light for 1 h to induce PIF7‐4MYC dephosphorylation, and then returned to white light for different durations to allow PIF7‐4MYC re‐phosphorylation. The ratio of phosphorylated to unphosphorylated PIF7‐4MYC was quantified to assess the phosphorylation dynamics. Consistent with the results in Figure 4B,C, higher levels of phosphorylated PIF7‐4MYC were detected in the *b″αβ* background than in the wild type following 1 h shade light treatment (Figure S10A). Quantitative analysis revealed that the rate of PIF7‐4MYC re‐phosphorylation was substantially higher in the *b″αβ* background (3.20/1.39 ≈ 3.2) than that in wild type (1.47/1 = 1.47) (Figure S10A). Together, these results indicate that loss of PP2A B″α and B″β leads to accumulation of phosphorylated PIF7‐4MYC.

To further strengthen our conclusion, we performed nucleo‐cytoplasmic fractionation of PIF7‐4MYC from *PIF7‐4MYC* overexpression lines in Col‐0 and *PIF7‐4MYC/4M* backgrounds grown under white light and shade light conditions. Figure S10B shows two distinct bands for PIF7‐4MYC corresponding to the phosphorylated (upper) and unphosphorylated (lower) forms under white light in both backgrounds in the total and cytoplasmic fractions. The lower band became more prominent under shade light in all three fractions, but especially in the nuclear fraction, indicating dephosphorylation of PIF7‐4MYC. Although, PIF7‐4MYC protein level appears to be lower in the *4M* background compared to Col‐0, the phosphorylated form of PIF7‐4MYC is more abundant under white light in the total and cytoplasmic fractions compared to nuclear fraction. Together, these findings highlight the highly dynamic nature of PIF7 phosphorylation and dephosphorylation in response to changing light environments.

### PP2A directly dephosphorylates PIF7 *in vitro*

PP2A has previously been shown to dephosphorylate PIF3‐MYC to regulate photomorphogenesis (Cai et al., 2024). To examine whether PP2A also directly dephosphorylates PIF7 to regulate shade responses, we performed the *in vitro* dephosphorylation assay. Immunoprecipitated complexes from *PP2A* overexpression lines served as the PP2A enzyme source, and supernatants from white light‐grown seedlings of PIF7‐FLASH served as substrates. Calf intestinal phosphatase (CIP) and boiled CIP treatments were included as positive and negative controls, respectively. Immunoprecipitated complexes from *35S:RCN1‐GFP, 35S:A3‐GFP, 35S:YFP‐B′α*, and 3*5S:YFP‐B′β* subunit overexpression lines efficiently dephosphorylated PIF7‐FLASH (Figure 4D; Figure S11), comparable to the CIP treatment positive control. In contrast, the immunoprecipitated material from Col‐0 failed to dephosphorylate PIF7‐FLASH. These results demonstrate that immunoprecipitated PP2A complexes can directly dephosphorylate PIF7‐FLASH *in vitro*.

### PP2A is required for PIF7 target gene expression under shade

**Figure 5:**
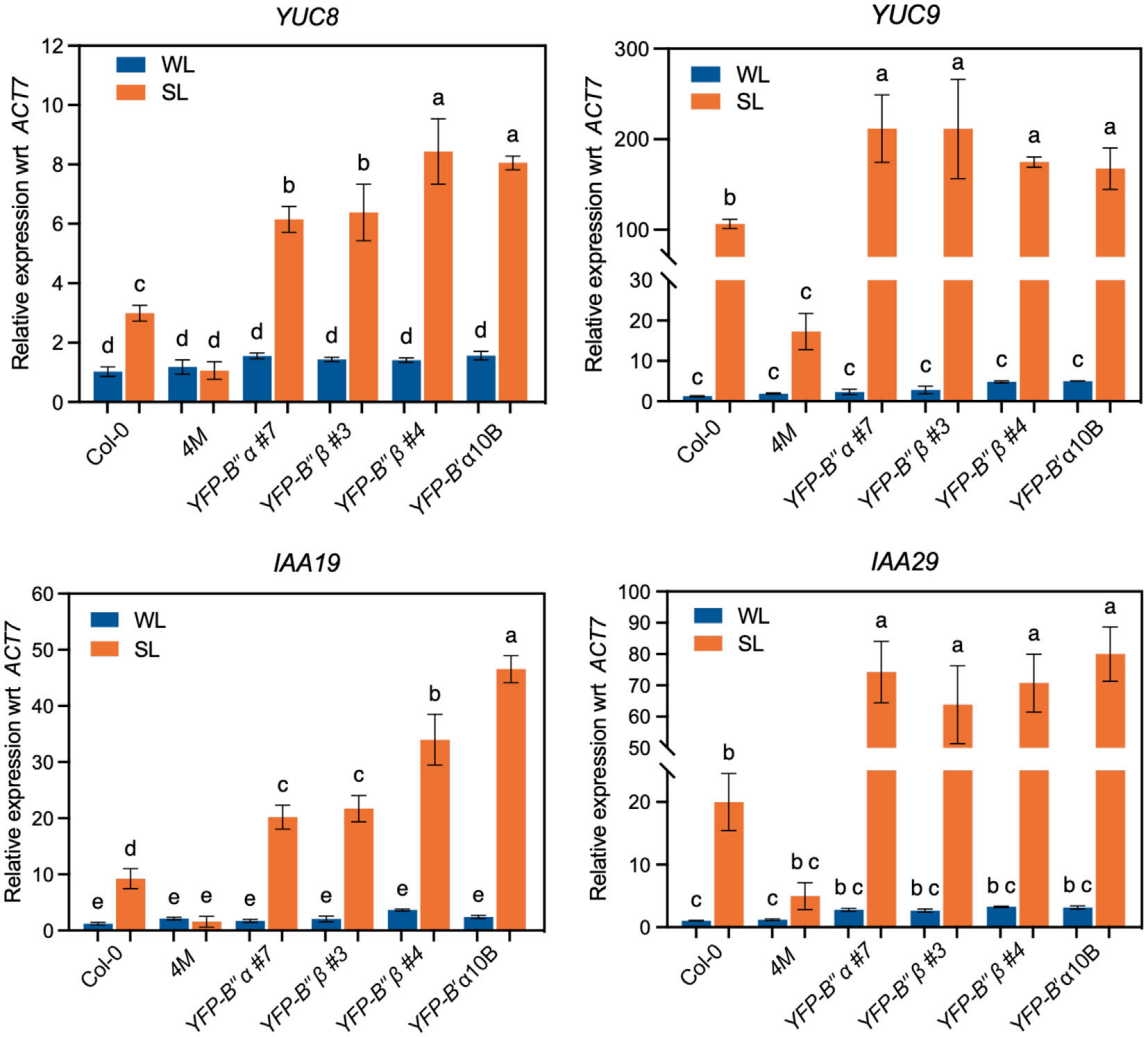
PP2A regulates PIF7 target gene expression under shade light: RT‐qPCR analysis using *YUC8*, *YUC9*, *IAA19* and *IAA29*. RT‐qPCR samples were extracted from 4 day white light (WL)‐grown seedlings of Col‐0, *pp2a b″αβ/b′αβ* quadruple mutant (*4M*), *YFP‐B′α*, *YFP‐B′β*, *YFP‐B″α*, and *YFP‐B″β* overexpression lines, and then were kept in white light or shade light for 1 h. Three biological repeats were performed. The error bars represent SEM (*n* = 3), Relative gene expression levels were normalized using the expression level of *ACT7* and the values of those genes in Col‐0 (WL). Statistically significant differences are indicated by different lowercase letters (*P* < 0.05), based on one‐way anova followed by Tukey's multiple comparison test. SL, shade light; WL, white light.

To examine whether PP2A affects PIF7 target gene expression, we performed RT‐qPCR analyses using wild type, quadruple mutant (*4M*) and B subunit overexpression seedlings grown under white and shade light conditions. *YUCCA8(YUC8)*, *YUCCA9(YUC9)*, *IAA19* and *IAA29* were selected as representative PIF7 target genes. In the wild type background, transcript levels of all four genes were strongly induced by shade treatment (Figure 5). In contrast, shade‐induced upregulation of these genes was markedly attenuated in the *4M* under shade conditions (Figure 5). Conversely, expression of all four genes was higher in the four B subunit overexpression lines under shade conditions, indicating that PP2A activity is required for PIF7‐mediated transcriptional activation during shade responses.

To assess whether expression of *PP2A* subunits is regulated by shade light, we performed RT‐qPCR analysis of PP2A B′ and B″ subunit transcripts. Expression of *B′α* and *B″β* showed a modest increase under shade conditions, whereas transcript levels of other PP2A subunits examined did not differ significantly between white and shade light (Figure S12A). To further examine whether shade affects PP2A subunit stability, 4‐day‐old white light‐grown seedlings were exposed to shade light for 1 h, and protein abundance was analyzed using an anti‐GFP immunoblotting. No significant changes in the protein levels of B′α, B′β, B″α, and B″β were detected following shade treatment (Figure S12B–I). Together, these results suggest that shade light has little, if any, effect on the expression or stability of the PP2A B′ and B″ subunits, and that PP2A promotes shade‐induced transcriptional responses primarily through post‐translational regulation of PIF7.

## DISCUSSION

Reversible phosphorylation of proteins, particularly transcription factors, by kinases and phosphatases plays a fundamental role in regulating cellular functions. This dynamic post‐translational modification provides an efficient mechanism to control protein activity, abundance, and subcellular localization (Johnson, 2009). Members of the basic helix–loop–helix (bHLH) transcription factor family, PHYTOCHROME‐INTERACTING FACTORs (PIFs), are extensively regulated by phosphorylation and dephosphorylation to coordinate diverse physiological responses, including shade‐avoidance syndrome (SAS) (Cai & Huq, 2025; Huq et al., 2024; Leivar & Monte, 2014). In most cases, phosphorylation of PIFs promotes their turnover through the 26S proteasome pathway (Huq et al., 2024). PIF7, however, exhibits a distinctive regulatory behavior: under white light, PIF7 exists in both phosphorylated and unphosphorylated forms, and upon exposure to shade, it is quickly dephosphorylated to a transcriptionally active form that induces the expression of auxin biosynthetic and signaling genes, thereby promoting SAS. Despite the identification of multiple kinases and phosphatases that have been reported to regulate other PIF family members (Cai & Huq, 2025), the enzymes responsible for PIF7 dephosphorylation have remained unknown. Here, we identify PP2A as a key phosphatase that regulates PIF7 activity.

**Figure 6:**
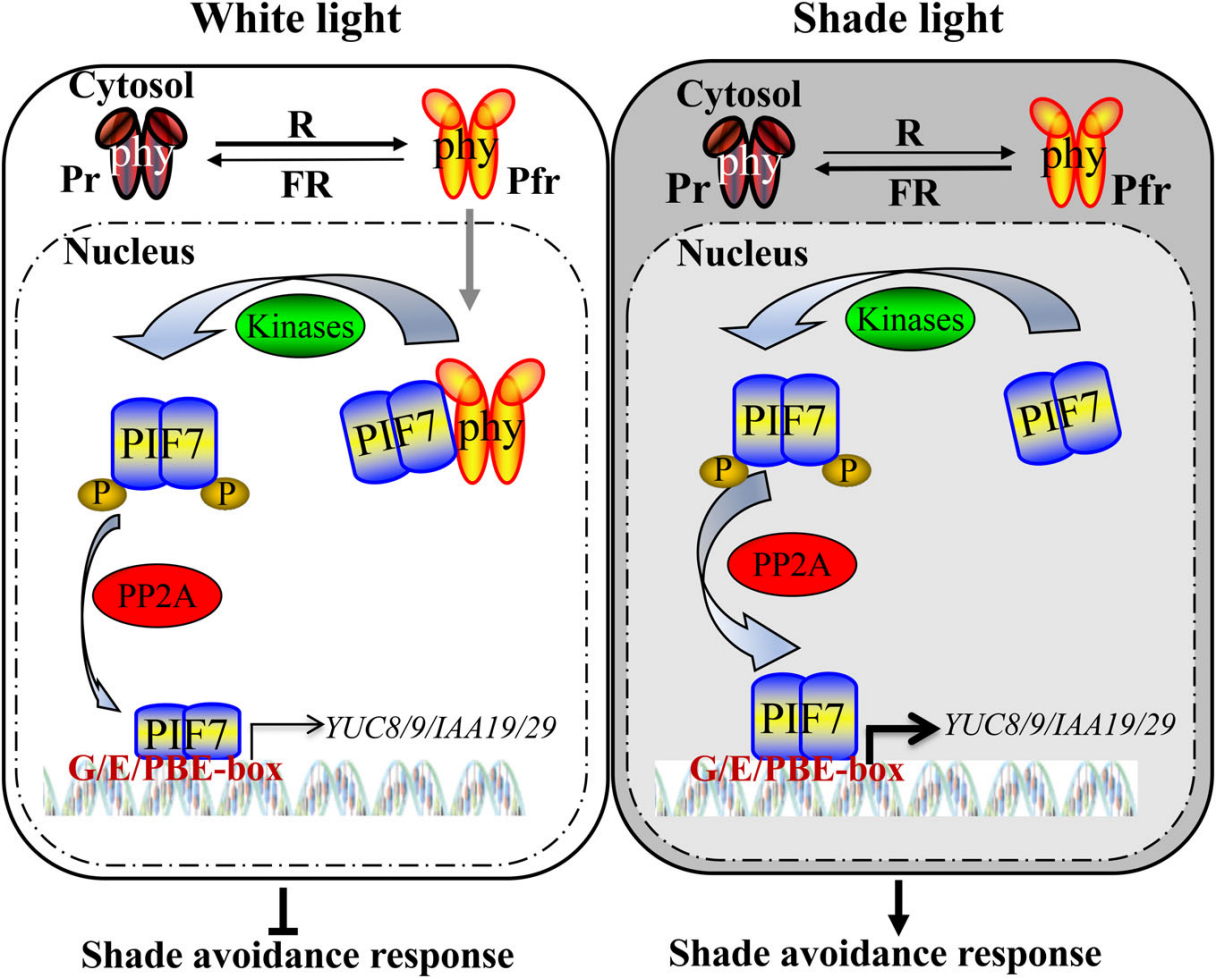
Model of PP2A‐mediated PIF7 dephosphorylation under shade conditions. Left panel: under white light, phytochromes (phy) predominantly exist in the active Pfr form and translocate into the nucleus, where they promote phosphorylation of PIF7 by unknown kinases. Phosphorylated PIF7 remains inactive and shows reduced binding to G/E/PBE‐box–containing promoters of shade‐responsive genes such as *YUC8/9* and *IAA19/29*, thereby limiting the shade‐avoidance response. Right panel: under shade light, the R/FR ratio decreases, shifting phytochromes toward the inactive Pr form and reducing phy‐mediated PIF7 phosphorylation. Protein phosphatase PP2A further dephosphorylates PIF7, enabling accumulation of the dephosphorylated, active form of PIF7 in the nucleus. Activated PIF7 binds to G/E/PBE‐box motifs and induces transcription of key shade‐responsive genes, thereby promoting the shade‐avoidance response.

Our genetic and biochemical analyses support a model in which PP2A B′α, B′β, B″α and B″β regulate SAS by dephosphorylating PIF7 (Figure 6). Loss‐of‐function *b″αβ* double and *4M* quadruple mutants display reduced hypocotyl elongation under shade conditions (Figure 1; Figure S1), suggesting that these four B subunits are required for a full shade‐avoidance response. Epistasis analyses further show that *pif7* is epistatic to *pp2a b* subunit mutants (Figure 3; Figure S6). Consistent with this genetic relationship, all four regulatory subunits interact with PIF7 in yeast two‐hybrid, *in vitro* pull‐down and *in vivo* co‐IP assays (Figure 2). In the absence of PP2A B″α and B″β, the phosphorylated form of PIF7 accumulates to higher levels compared with the wild type under shade conditions (Figure 4; Figure S10A). Pharmacological inhibition of PP2A activity using cantharidin, a well‐characterized inhibitor of PP1 and PP2A (Honkanen, 1993), stabilizes phosphorylated PIF7 under shade conditions (Figure 4), supporting a direct role for PP2A in PIF7 dephosphorylation. Moreover, immunopurified PP2A A and B subunits directly dephosphorylated PIF7 *in vitro* (Figure 4D; Figure S11). In agreement with these biochemical observations, PP2A B subunits positively regulate the expression of PIF7 target genes under shade conditions (Figure 5). Together, these results establish that PP2A plays an essential role in regulating SAS by controlling PIF7 phosphorylation status.

As a master regulator of shade responses, PIF7 is subject to multilayered regulation at the levels of protein stability, subcellular localization, DNA‐binding activity and chromatin context. For example, UBP12 and UBP13 enhance PIF7 function by stabilizing the protein under shade conditions (Zhou et al., 2021). HFR1, a non‐DNA‐binding HLH factor, heterodimerizes with PIF7 and prevents its association with target genes (Paulišić et al., 2021). In addition, the long non‐coding RNA PHYA UTR Antisense RNA (PUAR) physically associates with PIF7, resulting in inhibition of the shade‐induced expression of *PHYA* by blocking the binding of PIF7 to the 5′‐UTR of *PHYA* (Zhu et al., 2023). Circadian clock components, including ELF3, PRR5 and PRR7, also physically interact with PIF7 and gate shade responses by repressing PIF7‐dependent transcription at specific times of day (Jiang et al., 2019; Zhang et al., 2020). At the subcellular level, phosphorylated PIF7 is retained in the cytosol through interaction with 14‐3‐3 proteins, whereas shade‐induced dephosphorylation of PIF7 promotes its nuclear accumulation and transcriptional activity (Huang et al., 2018). Furthermore, epigenetic regulators such as ASF1‐HIRA and MRG1/2 interact with PIF7 and modulate target gene expression through chromatin‐based mechanisms (Peng et al., 2017; Yang et al., 2023). Our findings add PP2A B subunits to the growing regulatory network, revealing a unique mode of PIF7 regulation through direct dephosphorylation. Given that PP2A B subunits localize to both cytosol and nucleus (Cai et al., 2024), PP2A may regulate PIF7 in both locations to facilitate nuclear translocation and DNA binding.

In summary, PP2A B subunits represent new players of the shade‐avoidance signaling network in Arabidopsis. By directly interacting with and dephosphorylating PIF7, PP2A fine‐tunes transcriptional response to vegetative shade (Figure 6). Although *pp2a* double and quadruple mutants display short hypocotyl phenotype under shade, the phenotypic effects are relatively modest. There are at least 17 PP2A B subunits in Arabidopsis (Booker & DeLong, 2017; Farkas et al., 2007), suggesting that functional redundancy among family members may buffer the loss of individual subunits. Further studies will be required to determine whether additional PP2A B subunits contribute to the regulation of shade responses.

### Study limitations

Although PIF7 is rapidly dephosphorylated upon exposure to shade, PP2A B subunits interact with PIF7 under both white light and shade conditions, suggesting that PP2A activity toward PIF7 is not specific to shade. Consistent with this, the expression and stability of the PP2A B subunits analyzed here are not regulated by shade. Given the highly dynamic nature of PIF7 phosphorylation and dephosphorylation (Figure 4) (Li et al., 2012; Zhou et al., 2021), it is possible that PP2A functions under both light conditions. Alternatively, PP2A activity may remain relatively constant under both white light and shade conditions, the kinase activity may be reduced under shade, consequently leading to increased levels of dephosphorylated (active) PIF7. Moreover, under white light PIF7 activity is inhibited by phyB‐PIF7 condensate formation, while under shade light, phyB is inactivated to Pr form releasing PIF7 to control downstream gene expression for promoting hypocotyl elongation (Xie et al., 2023). Thus, PIF7 has a more prominent role in regulating hypocotyl length under shade compared to white light conditions. Therefore, PP2A‐mediated dephosphorylation of PIF7 has a more pronounced effect on PIF7 under shade compared to white light conditions. Further studies are necessary to dissect how PIF7 dephosphorylation has a greater impact under shade compared to white light conditions.

## MATERIALS AND METHODS

### Plant growth conditions and hypocotyl phenotype analysis

All the seeds are of *Arabidopsis thaliana* in Columbia‐0 (Col‐0) background. The T‐DNA insertion mutants used in this paper: *pp2ab″α* (SALK_135978), *b″β* (SALK_151964), and *b′αβ* (Tang et al., 2011). *B*″*αβ* has been described (Cai et al., 2024). These two lines (*b′αβ and b*″*αβ*) were crossed to generate *4M*. Generation of *B′α‐OX*, *B′β‐OX*, *B″α‐OX*, *B″β‐OX*, *RCN1‐OX*, *A3‐OX* transgenic plants has been described previously (Cai et al., 2024). *YFP‐B′α* and *YFP‐B′β* transgenic plants have also been previously described (Tang et al., 2011). For the *35S:PIF7‐4MYC* transgenic plants: PIF7 CDS was cloned into pENTR vector. Then, pENTR‐PIF7 was recombined to the pGWB17 gateway binary vector with the 35S promoter and c‐MYC tag by using LR Clonase II (Thermo Fisher Scientific, Cat# 11791020). PIF7‐4MYC destination vectors were transformed into the Col‐0, *b″αβ*, *pif7‐2, YFP‐B′α, YFP‐B′β*, *YFP*‐*B″α*, and *YFP*‐*B″β* backgrounds, and transformants were selected by using the Hygromycin antibiotic. *PIF7‐FLASH*, *pif7‐1*, and *pif7‐2* have been previously described (Li et al., 2012). For *pif7‐1/4M* and *pif7‐2/4M*, *4M* was crossed with *pif7‐1* and *pif7‐2*, respectively, and selected homozygous plants for phenotypic analysis. For *B′α‐OX/pif7‐2*, *B′α‐OX* was crossed with *pif7‐2* and then selected homozygous plants for phenotypic analysis. Seeds were surface sterilized in 1% (v/v) commercial bleach with 0.3% SDS for 10 min followed by five quick rinses with sterile water and then plated on Murashige and Skoog (MS) basal salts without sucrose. Where indicated, cantharidin was incorporated into the growth medium at a final concentration of 10 μm. Seeds were stratified at 4°C in the dark for 3 days followed by white light exposure for 3 days to promote germination and then either kept in the continuous white light (22°C) or continuous shade light treatment conditions (22°C) (White light PAR (400–700 nm) = 18.98 μmol·m^−2^·s^−1^ (R: FR = 9.7; including 5.2 μmol·m^−2^·s^−1^ red light, 0.53 μmol·m^−2^·s^−1^ far‐red, and 3.4 μmol·m^−2^·s^−1^ blue light); Shade light PAR (400–700 nm) = 19.18 μmol·m^−2^·s^−1^ (R:FR = 0.2; including 5.6 μmol·m^−2^·s^−1^ red light, 26.6 μmol·m^−2^·s^−1^ far‐red, 2.5 μmol·m^−2^·s^−1^ blue light)) for 6 days for Western blot analyses or hypocotyl elongation analyses. For the hypocotyl elongation analyses, 3 independent biological replicates with >20 seedlings were used in the measurements. Measurements were done using ImageJ software and analyzed using one‐way anova followed by Tukey's multiple comparison test. Statistical differences are indicated by different letters or asterisks (*P* < 0.05).

### Protein purification from *E. coli*

The MBP‐B′α, ‐B′β, ‐B″α and ‐B″β plasmids have been previously described (Cai et al., 2024). Each plasmid was transformed into BL21(DE3) cells. Protein expression was induced under 16°C for 12 h with 0.1 mm IPTG. Extraction buffer (50 mm Tris, pH 7.5, 150 mm NaCl, 1 mm EDTA, 0.1% Tween20, 0.25 mm DTT, 1× protease inhibitor cocktail, 1 mm PMSF) was added to the cell pellet and vortexed to resuspend the cells. Sonication was performed to break the cells, and the extracts were cleared by centrifugation at 20 000 ***g*** for 15 min. The supernatants were incubated with amylose resin (NEB, Cat#, E8021S) for 1 h in the dark at 4°C. Amylose resin was washed with extraction buffer three times for 10 min each time. The MBP protein was still bound to the resin for the following *in vitro* pull‐down assays.

### Protein interaction assays

For semi‐*in vivo* pull‐down assays, 5‐day‐old white light‐grown seedlings of *35S:PIF7‐FLASH* (Li et al., 2012) were pre‐treated with 100 μm bortezomib for 4 h in the dark. One batch was ground in liquid nitrogen. Another batch was exposed to shade light (R:FR = 0.2) for 20 min before being ground in liquid nitrogen. Total protein was solubilized in extraction buffer (50 mm Tris, pH 7.5, 150 mm NaCl, 1 mm EDTA, 0.1% Tween‐20, 0.25 mm DTT, 1× protease inhibitor cocktail (Sigma‐Aldrich, Cat# P9599), 1 mm PMSF). The extracts were cleared by centrifugation at 20 000 ***g*** for 15 min. The supernatants were incubated with beads bound with MBP‐B″α, MBP‐B″β, and MBP only as a control, respectively for 1 h in the dark at 4°C. Beads were washed three times, 10 min each with extraction buffer. Beads were then boiled with 2× SDS sample buffer and the supernatants were separated on an SDS–PAGE gel. Anti‐MYC antibody was used to detect PIF7‐FLASH protein.

For yeast two‐hybrid assay, AD plasmids (AD‐PIF7), and BD plasmids (BD‐B′α, BD‐B′β, BD‐B″α, BD‐B″β) were transformed into the AH109 yeast cell simultaneously and then selected on a solid medium lacking Leu and Trp amino acids (−LT). Only successfully transformed yeast cells survive on −LT medium. Then these yeast cells were plated on a solid medium lacking Leu, Trp, and His amino acids (−LTH) and medium lacking Ade, Leu, Trp, and His amino acids (−ALTH). The synthesis of His and Ade can be activated when B′α, B′β, B″α and B″β interact with PIF7 and then the yeast can survive on the −LTH and −ALTH medium. To reduce background and/or avoid false‐positive results, 3‐amino‐1,2,4‐triazole (3‐AT) was added to the −LTH medium to inhibit the self‐activation of the His synthesis.

For the *in vivo* co‐IP assays, 4‐day‐old white light‐grown (R:FR = 9.7) *35S:YFP‐B″α/PIF7‐4MYC*, *35S:YFP‐B″β/PIF7‐4MYC*, *35S:YFP‐B′α/PIF7‐4MYC*, *35S:YFP‐B′β/PIF7‐4MYC*, *35S:YFP‐B″α*, *35S:PIF7‐4MYC*, and Col‐0 seedlings were used. *35S:YFP‐B″α*, *35S:PIF7‐4MYC* and Col‐0 were used as controls. The seedlings were pre‐treated with 100 μm bortezomib for 4 h in darkness. One batch was ground in liquid nitrogen. Another batch was exposed to shade light (R:FR = 0.2) for 20 min before being ground in liquid nitrogen. Total protein was solubilized in an extraction buffer [50 mm Tris, pH 7.5, 150 mm NaCl, 1 mm EDTA, 0.2% NP40 (v/v), 0.25 mm DTT, 1× protease inhibitor cocktail, 1 mm PMSF, 10 mm NaF, 2 mm Na orthovanadate]. The extracts were cleared by centrifugation at 20 000 ***g*** for 15 min. The supernatants were incubated with Dynabeads bound with anti‐MYC (Cell Signaling, Cat#2278S) for 1 h in the dark at 4°C. The Dynabeads were washed with the wash buffer [50 mm Tris, pH 7.5, 150 mm NaCl, 1 mm EDTA, 1% NP40 (v/v), 0.25 mm DTT, 1× protease inhibitor cocktail, 1 mm PMSF, 10 mm NaF, 2 mm Na orthovanadate] 3 times for 10 min each time. The beads were heated to 65°C for 15 min with a 2× SDS sample buffer and separated on a 10% SDS–PAGE gel. An anti‐GFP antibody (Invitrogen‐Thermofisher, Cat#MA5‐15256; dilution 1:5000) was used to detect the YFP‐B″α, YFP‐B″β, YFP‐B′α, YFP‐B′β proteins. Anti‐MYC antibody (Cell Signaling, Cat#2278S; dilution 1:10 000) was used to detect the PIF7‐MYC proteins.

### PIF7 phosphorylation status assay

Five‐day‐old white light‐grown seedlings of *35S:PIF7‐4MYC* in Col‐0 and *b″αβ* were pre‐treated with 100 μm bortezomib and 50 μm MG132 for 4 h, then treated with shade light (R:FR = 0.2) for different times or just kept in white light (R:FR = 9.7) conditions before being ground in liquid nitrogen. Samples were then boiled with 2× SDS sample buffer and the supernatants were separated on an SDS–PAGE gel (8% gel). Anti‐MYC antibody (Cell Signaling, Cat#2278S; dilution 1:10 000) was used to detect PIF7‐4MYC protein.

### Nucleo‐cytoplasmic fractionation of PIF7‐4MYC

Nuclear and cytosolic fractions were isolated following the protocol described (Zhao et al., 2022). Six‐day‐old white light‐grown seedlings were either maintained under white light or transferred to shade light (SL) conditions for 1 h prior to harvest. Seedlings were immediately frozen in liquid nitrogen, ground to a fine powder, and homogenized in Honda buffer (25 mm Tris–HCl pH 7.4, 10 mm MgCl_2_, 2.5% Ficoll‐400, 250 mm sucrose, 0.5% Triton X‐100, 10 mm β‐mercaptoethanol, 0.5 mm PMSF, 1× protease inhibitor cocktail). The homogenate was filtered twice through Miracloth (40 μm filter‐ CalBiochem), and 100 μl of the filtrate was collected as the total protein fraction. The remaining filtrate was centrifuged at 1500 ***g*** for 5 min at 4°C to pellet nuclei and cellular debris. From the resulting supernatant, 100 μl was collected and further centrifuged at 12 000 ***g*** for 10 min at 4°C; the clarified supernatant was designated as the cytosolic fraction. The pellet obtained from the prior centrifugation step was subjected to sequential washes with nuclei isolation buffer (20 mm KCl, 20 mm HEPES pH 7.4, 0.5% v/v Triton X‐100, 13.8% v/v hexylene glycerol, 0.1% v/v β‐mercaptoethanol, 50 μm spermine, 125 μm spermidine, 1 mm PMSF, 1× protease inhibitor cocktail) until the pellet appeared white (typically 3 to 4 washes). The purified nuclear pellet was resuspended in 30 μl of pre‐chilled glycerol buffer (20 mm Tris–HCl pH 8.0, 50% v/v glycerol, 75 mm NaCl, 0.5 mm EDTA, 0.85 mm DTT, 0.125 mm PMSF, 1× protease inhibitor cocktail) and combined with an equal volume (30 μl) of nuclei lysis buffer (10 mm HEPES pH 7.6, 1 mm DTT, 7.5 mm MgCl_2_, 0.2 mm EDTA, 0.3 m NaCl, 1 m urea, 1% v/v NP‐40, 0.5 mm PMSF, 1× protease inhibitor cocktail). The mixture was incubated in ice for 10 min to achieve complete nuclear lysis, and the resulting lysate was used as the nuclear fraction. All the fraction samples were boiled with SDS loading dye before loading on the gel. Anti‐Myc antibody (Cell Signaling, Cat#2278S; dilution 1:10 000) was used to detect PIF7‐4MYC protein, Anti‐Histone 3 (Abcam, Cat#ab1791; dilution 1:10 000) and Anti‐Tubulin (Sigma aldrich, Cat#T5168; dilution 1:10 000) antibodies were used to probe the nuclear fraction control and cytosolic control, respectively.

### PIF7 dephosphorylation assay

PIF7‐FLASH extracts: 5‐day‐old white light‐grown seedlings of *35S:PIF7‐FLASH* were pre‐treated with 100 μm bortezomib and 50 μm MG132 for 4 h before being ground in liquid nitrogen. The sample was ground in the buffer (50 mm Tris‐Cl (pH = 7.5), 150 mm NaCl, 1% Triton X‐100, 1 mm PMSF, 100 μm bortezomib, 1× protease inhibitor cocktail, 25 mm β‐glycerophosphate, 10 mm NaF, and 2 mm Na orthovanadate). After centrifugation, the supernatants of PIF7‐FLASH were aliquoted into different microtubes as substrate.

IP for PP2A: 4‐day‐old dark‐grown seedlings of *35S:RCN1‐GFP*, *35S:A3‐GFP*, *35S:YFP‐B′α*, *35S:YFP‐B′β* and Col‐0 (as negative control) were ground in the buffer (50 mm Tris‐Cl (pH = 7.5), 150 mm NaCl, 0.1% NP‐40, 1 mm PMSF, 100 μm bortezomib, 1 × protease inhibitor cocktail, 5 mm CaCl_2_). Samples were immunoprecipitated using anti‐GFP (Abcam, Cat. # ab290) antibody pre‐bound to Dynabeads. IP products were washed once by wash buffer (50 mm Tris‐Cl (pH = 7.5), 150 mm NaCl, 0.1% NP‐40, 5 mm CaCl_2_).

Dephosphorylation: Mix the IP products from *35S:RCN1‐GFP, 35S:A3‐GFP, 35S:YFP‐B′α, 35S:YFP‐B′β* and Col‐0 with PIF7‐FLASH substrate, respectively, in the buffer (50 mm Tris‐Cl (pH = 7.5), 150 mm NaCl, 10 mm MgCl_2_, 1 mm MnCl_2_, 1 mm DTT, 1 × protease inhibitor cocktail, 5 mm CaCl_2_), incubate in 30°C shaker for 1 h. Also mix Quick CIP (NEB, Cat. #M0525) or boiled Quick CIP with the substrate, respectively, in the buffer (50 mm Tris‐Cl (pH = 7.5), 100 mm NaCl, 10 mm MgCl_2_, 1 mm DTT, 1× protease inhibitor cocktail), incubate at 37°C for 1 h. Western blot was then performed by using 7% gel, and anti‐MYC (Cell Signaling, Cat#2278S; dilution 1:10 000) was used to detect PIF7‐FLASH signals.

### RNA isolation and RT‐qPCR

Four‐day‐old white light‐grown seedlings were used with 3 independent biological replicates (*n* = 3). The seeds were surface sterilized and plated on MS media without sucrose, cold‐stratified for 3 days, and then kept under continuous white light. After 4 days, the seedlings were either kept in white light or exposed to shade light (R:FR = 0.2) for 1 h. Total RNA was isolated using the plant total RNA kit (Sigma, Cat# STRN250). For cDNA synthesis, 2 μg of total RNA was used for reverse transcription with M‐MLV Reverse Transcriptase (ThermoFisher Scientific, Cat# 28025013). A SYBR Green PCR master mix (ThermoFisher Scientific, Cat# 4368577) and gene‐specific oligonucleotides were used to conduct qPCR analyses using primers shown in Table S1. Finally, the relative transcription level was calculated using the 2^−ΔΔ*CT*^ method, by normalizing to *ACT7*.

## AUTHOR CONTRIBUTIONS

XC, OM and EH conceived the study and designed experiments; XC, OM and AG performed experiments; WT and YS provided materials; XC and OM wrote the manuscript; EH revised and edited the manuscript.

## CONFLICT OF INTEREST

The authors declare no conflict of interest.

## Supporting information


**Figure S1.** PP2A positively regulates shade light‐induced hypocotyl elongation.
**Figure S2.** PP2A positively regulates shade light‐induced hypocotyl elongation.
**Figure S3.** PIF7 does not interact with PP2A A subunits.
**Figure S4.** PP2A B″α and B″β enhance the long hypocotyl phenotype of *PIF7‐4MYC* under shade.
**Figure S5.** PIF7 interacts with PP2A B*″α* and B′*α in vivo*.
**Figure S6.** PP2A and PIF7 function in the same genetic pathway.
**Figure S7.** PP2A regulates dynamics of PIF7 phosphorylation–dephosphorylation.
**Figure S8.** PP2A regulates PIF7 phosphorylation status and activity during shade‐induced growth responses.
**Figure S9.** PP2A B″α and B″β regulate the long hypocotyl phenotype of *PIF7‐4MYC* under shade.
**Figure S10.** PP2A B subunits regulate PIF7 phosphorylation status and subcellular partitioning under shade.
**Figure S11.** PP2A can dephosphorylate PIF7‐FLASH *in vitro*.
**Figure S12.** Gene Expression level and protein accumulation of PP2A subunits under shade light condition.
**Table S1.** Primers used in this study.

## Data Availability

All data supporting the findings of this study are available within the manuscript and its Supporting Information. Arabidopsis mutants and transgenic lines, as well as plasmids and antibodies generated during this study, are available from the corresponding author upon reasonable request. No new sequencing data were created or analyzed in this study.
