# Supplementary material for: PP2A regulates shade avoidance response by dephosphorylating phytochrome‐interacting factor 7 in Arabidopsis
Source: Plant J. 2026 Aug 2;127(3):e71073. doi: 10.1111/tpj.71073 (PMC13428991; doi:10.1111/tpj.71073)
Supplement: Supplementary file 1 — Figure S1. PP2A positively regulates shade light‐induced hypocotyl elongation. Figure S2. PP2A positively regulates shade light‐induced hypocotyl elongation. Figure S3. PIF7 does not interact with PP2A A subunits. Figure S4. PP2A B″α and B″β enhance the long hypocotyl phenotype of PIF7‐4MYC under shade. Figure S5. PIF7 interacts with PP2A B″α and B′α in vivo. Figure S6. PP2A and PIF7 function in the same genetic pathway. Figure S7. PP2A regulates dynamics of PIF7 phosphorylation–dephosphorylation. Figure S8. PP2A regulates PIF7 phosphorylation status and activity during shade‐induced growth responses. Figure S9. PP2A B″α and B″β regulate the long hypocotyl phenotype of PIF7‐4MYC under shade. Figure S10. PP2A B subunits regulate PIF7 phosphorylation status and subcellular partitioning under shade. Figure S11. PP2A can dephosphorylate PIF7‐FLASH in vitro. Figure S12. Gene Expression level and protein accumulation of PP2A subunits under shade light condition. Table S1. Primers used in this study. [file TPJ-127-0-s001.pdf]

SUPPLEMENTARY INFORMATION

PP2A Regulates Shade Avoidance Response by Dephosphorylating Phytochrome-Interacting Factor 7 in *Arabidopsis*

Xingbo Cai, Oihik Mitra, Amariah Gustamante, Wenqiang Tang, Yu Sun and Enamul Huq

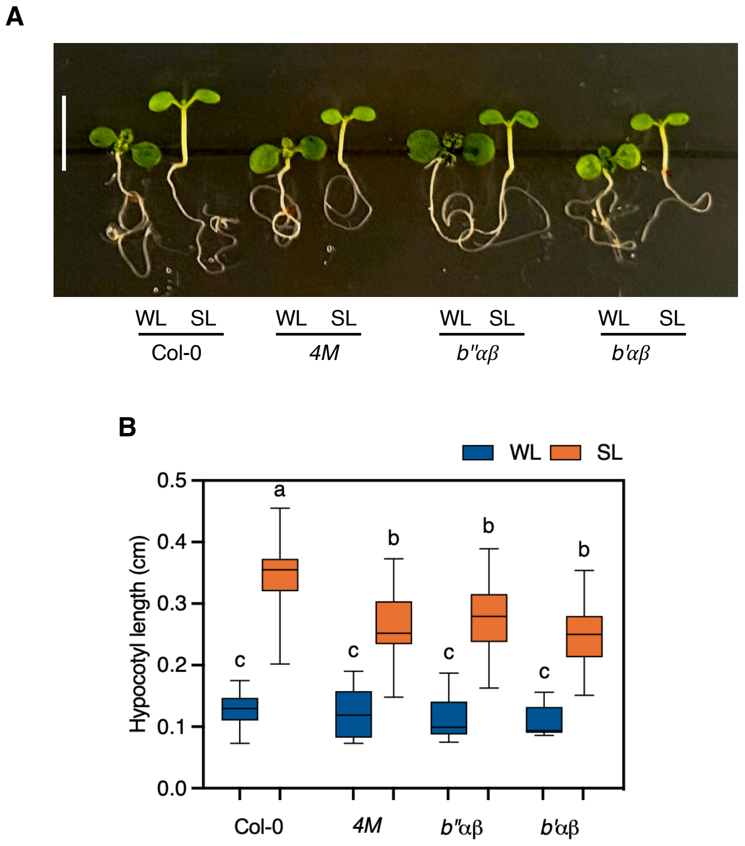

Figure S1: PP2A positively regulates shade light-induced hypocotyl elongation. Seedlings were grown under white light for 3 days to allow germination then either kept in the white light (22° C) or continuous shade light conditions for 6 days. **(A)** Photographs of seedlings showing hypocotyl length of *b''αβ, b'αβ and 4M* is shown compared to wild type (Col-0) under white light and shade light condition. **(B)** The boxplots exhibit the hypocotyl lengths of seedlings shown in **A.** Three biological replicates of >20 seedlings were used for measurements. The error bars represent SEM. A one-way ANOVA followed by Tukey's multiple comparison test was performed. Statistically significant differences are indicated by different lowercase letters ($P < 0.05$). WL, white light; SL, shade light.

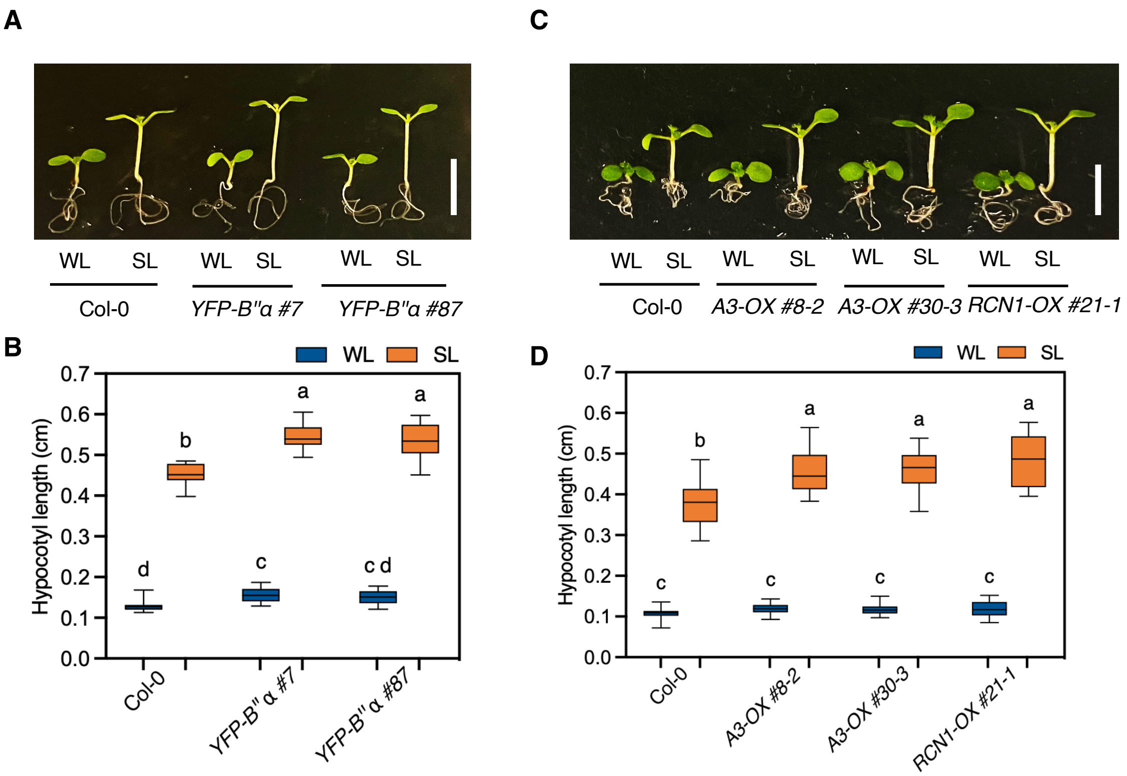

Figure S2: PP2A positively regulates shade light-induced hypocotyl elongation. Seedlings were grown under white light for 3 days to allow germination then either kept in the white light (22° C) or continuous shade light conditions for 6 days. **(A,C)** Photographs of seedlings showing hypocotyl length: The PP2A overexpression lines (*35S:YFP-B''α#7, 35S:YFP-B''α #87, 35S:A3-OX#8-2, 35S:A3-OX#30-3 and 35S:RCN1-OX#21-1)* hypocotyl length is shown compared to wild type (Col-0) under white light and shade light condition. **(B)** The boxplots exhibit the hypocotyl lengths of seedlings shown in **A. (D)** The boxplots exhibit the hypocotyl lengths of seedlings shown in **C**. Three biological replicates of >20 seedlings were used for measurements. The error bars represent SEM. A one-way ANOVA followed by Tukey's multiple comparison test was performed. Statistically significant differences are indicated by different lowercase letters ($P < 0.05$). WL, white light; SL, shade light.

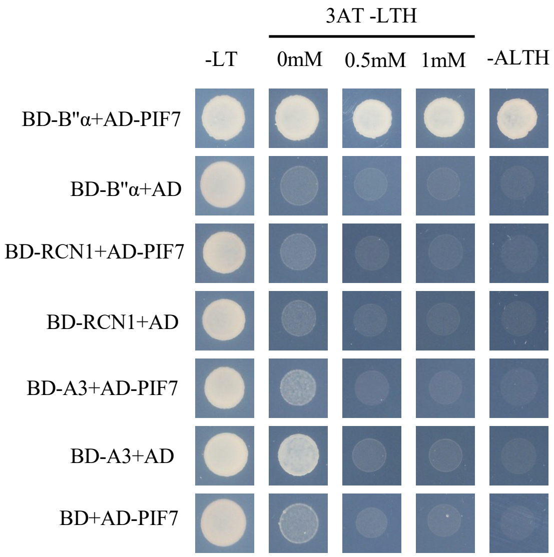

Figure S3: PIF7 does not interact with PP2A A subunits. Yeast two-hybrid (Y2H) assays showing the lack of interaction between full-length PIF7 and PP2A A subunits. The RCN1- and A3-GAL4 DNA-binding domain fusions (BD-RCN1 and BD-A3) were co-expressed with the GAL4 activation domain (AD) fused to full-length PIF7 or with AD alone as a negative control. Yeast cells were grown on selective media lacking histidine and supplemented with increasing concentrations of the histidine biosynthesis inhibitor 3-amino-1,2,4-triazole (3-AT). BD- B″α and AD-PIF7 were used as a positive control. –LT: medium lacking Leu and Trp amino acids; –LTH: medium lacking Leu, Trp, and His amino acids; -ALTH medium lacking Ade, Leu, Trp, and His amino acids.

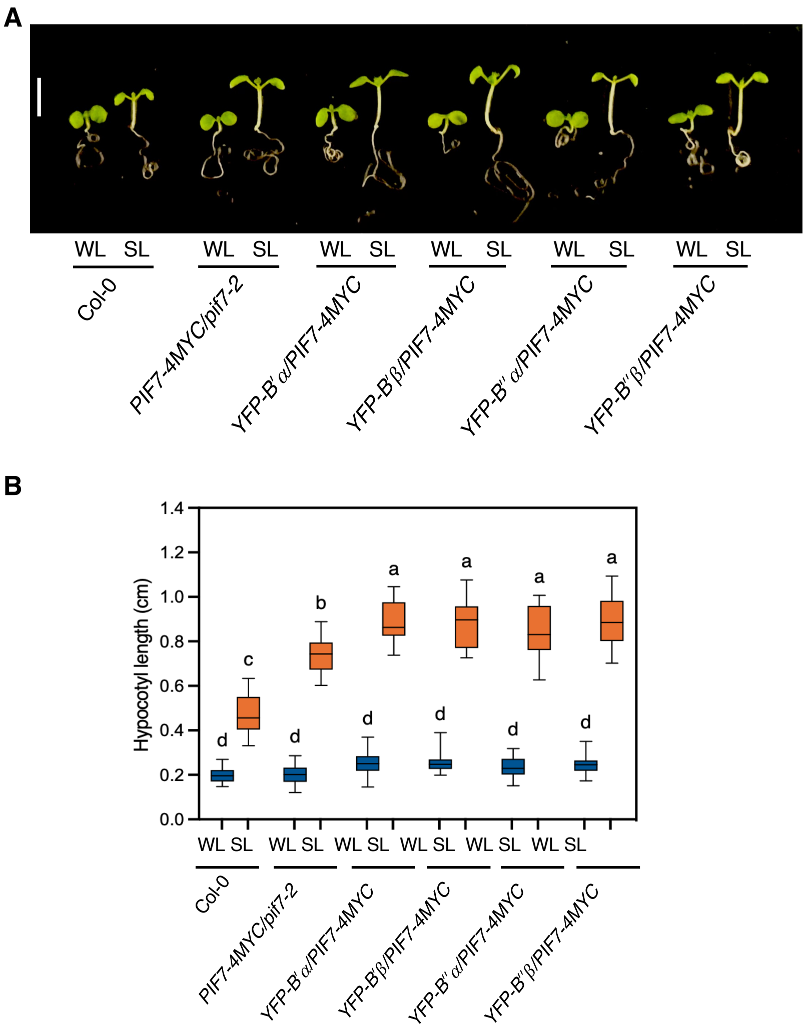

Figure S4: PP2A B′′α and B′′β enhance the long hypocotyl phenotype of *PIF7-4MYC* under shade. Seedlings were grown under white light for 3 days to allow germination then either kept in the white light (22° C) or continuous shade light conditions for 6 days. **(A)** Photographs of seedlings showing hypocotyl lengths of double overexpression lines (*35S:PIF7-4MYC/YFP-B'α, 35S:PIF7-4MYC/YFP-B'β, 35S:PIF7-4MYC/YFP-B"α,* and *35S:PIF7-4MYC/YFP-B"β)* compared to wild type (Col-0) and *35S:PIF7-4MYC/Col-0* backgrounds. **(B)** The boxplot exhibits the hypocotyl lengths of seedlings shown in **A**. Three biological replicates of >20 seedlings were used for measurements. The error bars represent SEM. A one-way ANOVA followed by Tukey's multiple comparison test was performed. Statistically significant differences are indicated by different lowercase letters (P < 0.05). WL, white light; SL, shade light.

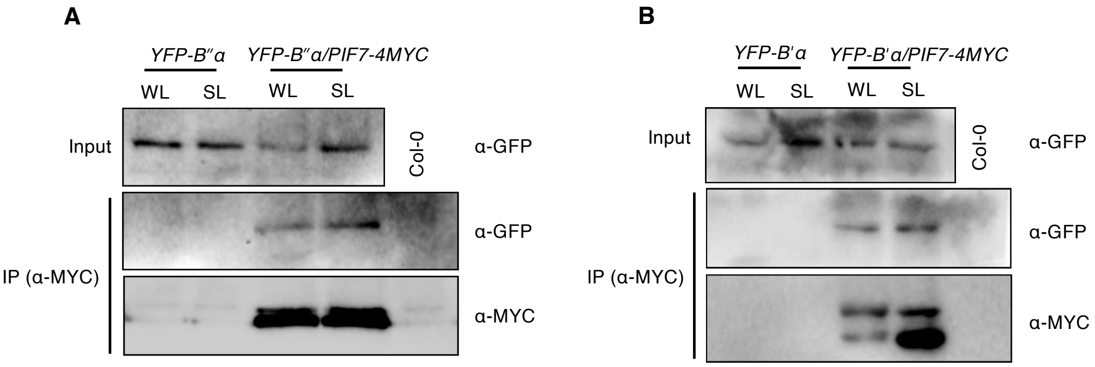

Figure S5. PIF7 interacts with PP2A B*''α* and B*'α in vivo*. (A-B) In vivo co-immunoprecipitation (co-IP) assays showing that PIF7-MYC interaction with B'α-GFP, B''α-GFP under both white-light and shade-light conditions. Four-day-old white-light grown seedlings of *35S:YFP-B''α, 35S:YFP-B''α/PIF7-MYC, 35S:YFP-B'α, 35S:YFP-B'α/PIF7-MYC* and Col-0 were used. *YFP-B''α, YFP-B'α* and Col-0 served as negative controls. One batch was maintained in white-light, and another was exposed to shade light treatment for 20 minutes. All seedlings were treated with 100μM Bortezomib for 4 h in darkness. α-MYC antibody was used for immunoprecipitation of PIF7-MYC and α-GFP antibody was used to detect YFP-B''α and YFP-B'α . WL, White light; SL, Shade light.

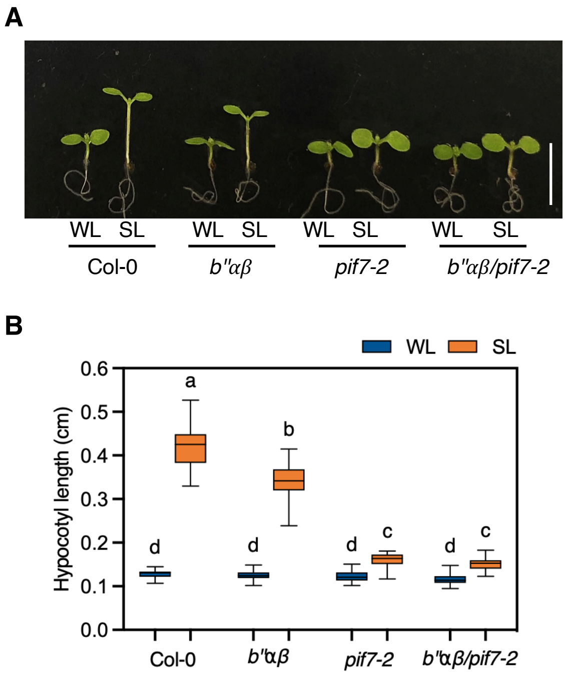

Figure S6: PP2A and PIF7 function in the same genetic pathway. Seedlings were grown under white light for 3 days to allow germination then either kept in the white light (22° C) or continuous shade light conditions for 6 days. **(A)** Photographs of seedlings showing hypocotyl length of *pp2ab″αβ/pif7-2* double transgenics is shown compared to wild type (Col-0), pif7-2 and pp2ab*″αβ* under white light and shade light condition. **(B)** The boxplots exhibit the hypocotyl lengths of seedlings shown in **A**. Three biological replicates of >20 seedlings were used for measurements. The error bars represent SEM. A one-way ANOVA followed by Tukey's multiple comparison test was performed. Statistically significant differences are indicated by different lowercase letters ($P < 0.05$). WL, white light; SL, shade light.

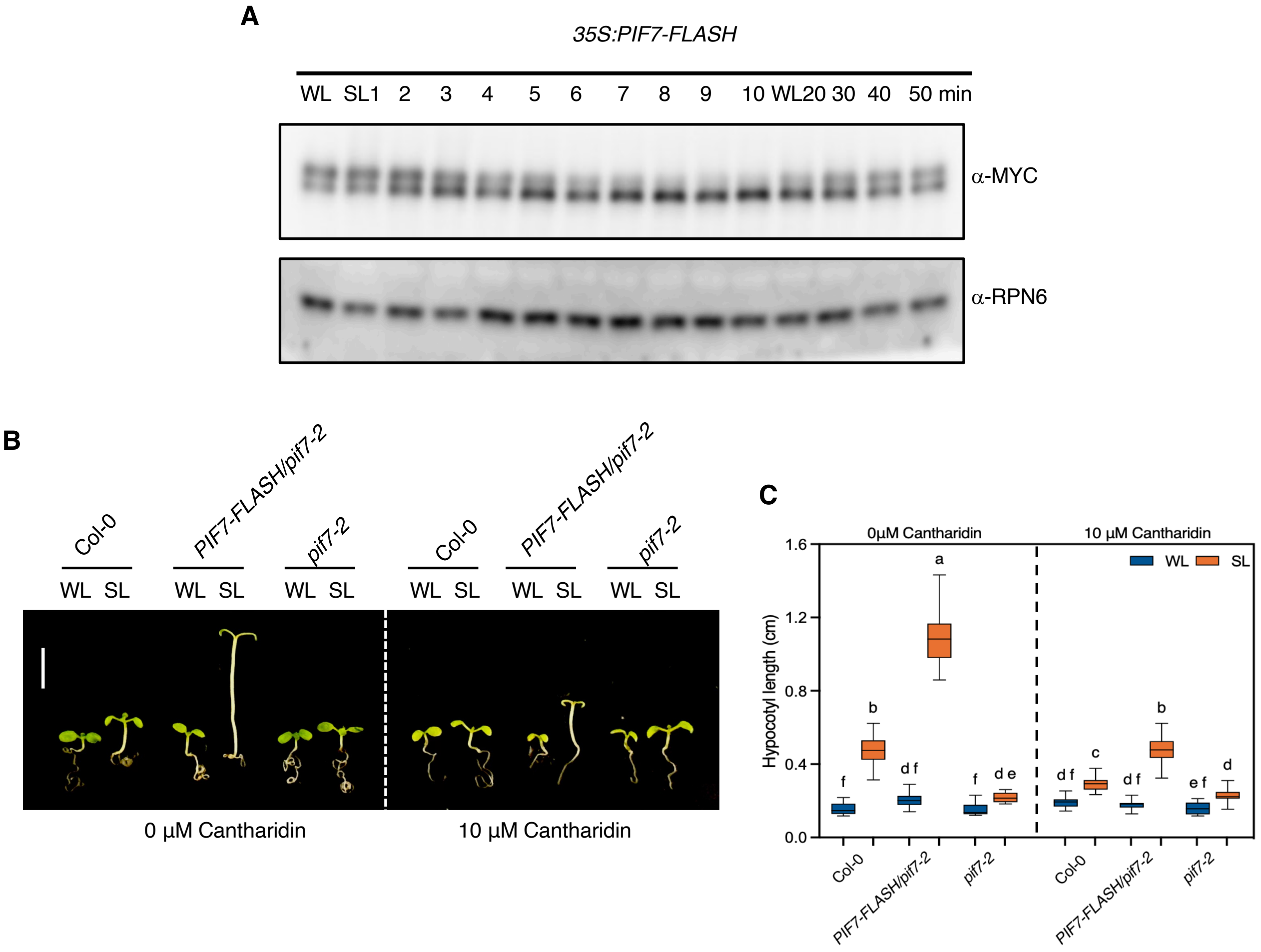

Figure S7: PP2A regulates dynamics of PIF7 phosphorylation-dephosphorylation. (A) Immunoblots showing the dynamic phosphorylation and dephosphorylation pattern of PIF7-FLASH under shade and white light conditions. Five-day-old white-light–grown *35S:PIF7-FLASH* seedlings were treated with shade light for the indicated durations and then returned to white light for various time periods. α-MYC antibody was used to detect PIF7-FLASH. **(B-C)** Col-0, *PIF7-FLASH,* and *pif7-2* seedlings were plated on MS-sucrose plates with or without 10 µM Cantharidine and grown under white light (WL) for 3 days to allow germination, then either kept in white light (22 °C) or transferred to continuous shade light for 6 days. Photographs of seedlings showing hypocotyl length **(B)**. **(C)** The boxplots exhibit the hypocotyl lengths of seedlings shown in **B**. Three biological replicates of >20 seedlings were used for measurements. The error bars represent SEM. A one-way ANOVA followed by Tukey's multiple comparison test was performed. Statistically significant differences are indicated by different lowercase letters ($P < 0.05$). WL, white light; SL, shade light.

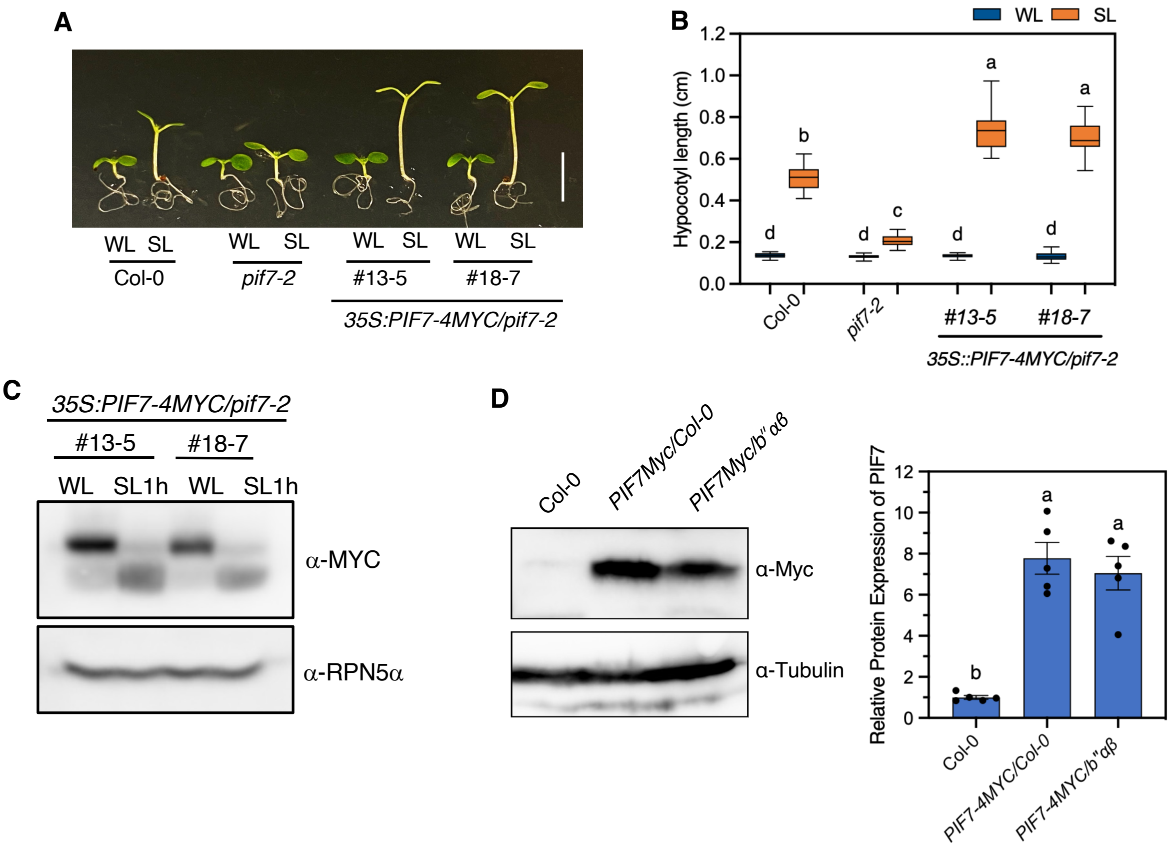

Figure S8: PP2A regulates PIF7 phosphorylation status and activity during shade-induced growth responses: (A) Photographs of seedlings showing hypocotyl length of two independent lines (#13-5 and #18-7) of *35S:PIF7-4MYC/pif7-2* is shown compared to wild type (Col-0) and *pif7-2* under white light and shade light conditions. **(B)** The boxplots exhibit the hypocotyl lengths of seedlings shown in **A**. **(C)** Immunoblots showing the PIF7-4MYC dephosphorylation pattern in two independent lines (#13-5 and #18-7) of *35S:PIF7-4MYC/pif7-2.* Five-day-old white-light–grown seedlings were pre-treated with 100µM Bortezomib and 100µM MG132 for 4h in darkness and then exposed to shade light for 1h. PIF7-4MYC was detected using α-MYC antibody. Upper and lower bands represent phosphorylated and unphosphorylated forms of PIF7-4MYC respectively, RPN5*α* was used as loading control. **(D)** (Left) Immunoblot shows the protein level of PIF7-4MYC in wild type Col-0 and *pp2ab″αβ* double mutant backgrounds. (Right) Quantitation of PIF7-4MYC levels in wild type Col-0 and *pp2ab″αβ* double mutant backgrounds from multiple independent blots (n=5). The error bars represent SEM. A one-way ANOVA followed by Tukey's multiple comparison test was performed. Statistically significant differences are indicated by different lowercase letters ($P < 0.05$). WL, white light; SL, shade light.

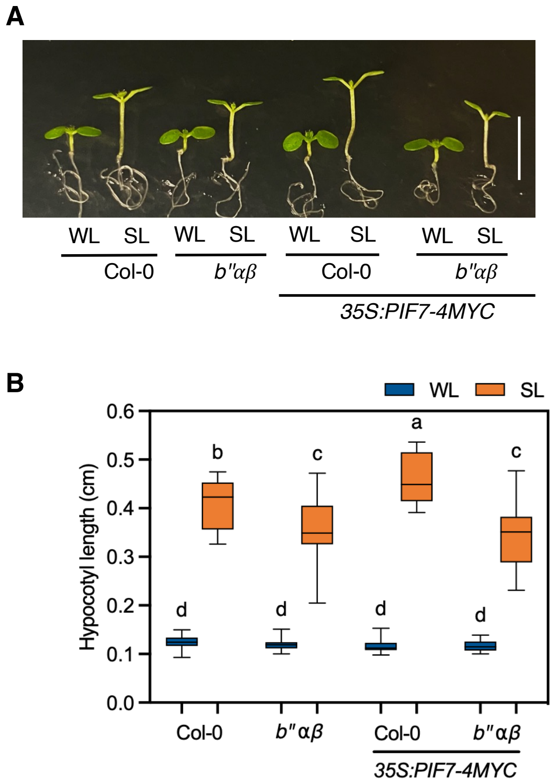

Figure S9: PP2A B′′α and B′′β regulate the long hypocotyl phenotype of *PIF7-4MYC* under shade. Seedlings were grown under white light for 3 days to allow germination then either kept in the white light (22° C) or continuous shade light conditions for 6 days. **(A)** Photographs of seedlings showing hypocotyl lengths of *35S:PIF7-4MYC/Col-0* and *35S:PIF7-4MYC/b"αβ* compared to wild type (Col-0) and pp2a *b"αβ.* **(B)** The boxplot exhibits the hypocotyl lengths of seedlings shown in **A**. **(C)** Photographs of seedlings showing hypocotyl lengths of double overexpression lines (*35S:PIF7-4MYC/YFP-B'α, 35S:PIF7-4MYC/YFP-B'β, 35S:PIF7-4MYC/YFP-B"α,* and *35S:PIF7-4MYC/YFP-B"β)* compared to wild type (Col-0) and *35S:PIF7-4MYC/Col-0* backgrounds. **(D)** The boxplot exhibits the hypocotyl lengths of seedlings shown in **C**. Three biological replicates of >20 seedlings were used for measurements. The error bars represent SEM. A one-way ANOVA followed by Tukey's multiple comparison test was performed. Statistically significant differences are indicated by different lowercase letters ($P < 0.05$). WL, white light; SL, shade light.

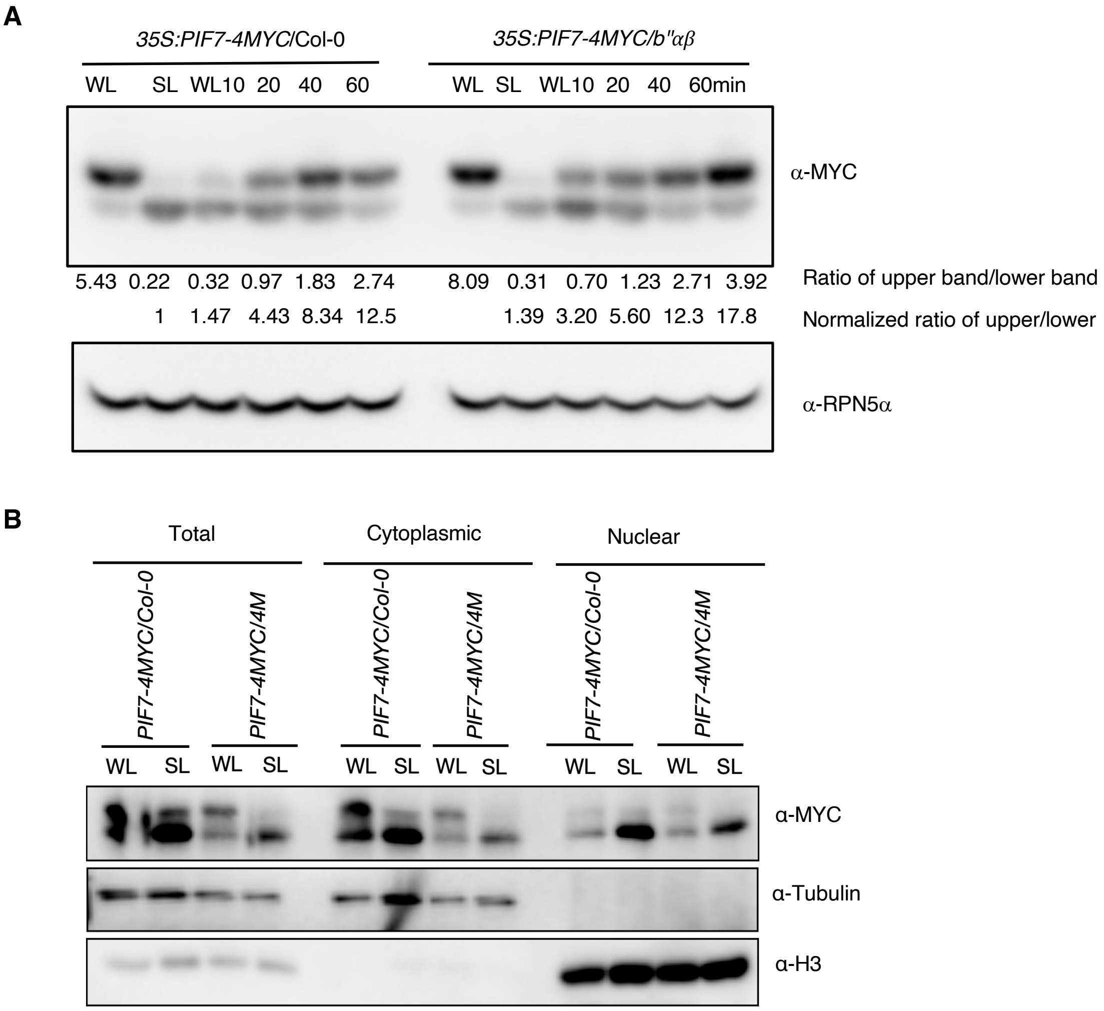

Figure S10: PP2A B subunits regulate PIF7 phosphorylation status and subcellular partitioning under shade. (A) Immunoblots showing the PIF7-4MYC dephosphorylation pattern in Col-0 and *pp2ab″αβ* (*b″αβ*) backgrounds. Five-day-old white-light–grown seedlings were pre-treated with 100μM Bortezomib and 100μM MG132 for 4 h in darkness and then exposed to shade light for 1h and then returned to white light for various time periods. PIF7-4MYC was detected using α-MYC antibody. Upper and lower PIF7-FLASH bands represent phosphorylated and unphosphorylated forms respectively, The upper/lower band ratio are shown below the blot α-MYC blot along with the normalized ratio relative to the loading control (RPN5α). WL, white light; SL, shade light. **(B)** Immunoblot showing the nucleo-cytoplasmic partitioning of PIF7-4MYC protein in wild type Col-0 and *pp2a 4M* backgrounds under white and shade light conditions. Seedlings were grown under white light for 3 days to allow germination then either kept in the white light (22°C) or continuous shade light conditions for 6 days. α-Tubulin and α-H3 antibodies show the purity of the nuclear and cytoplasmic fractions. WL, white light; SL, shade light.

A

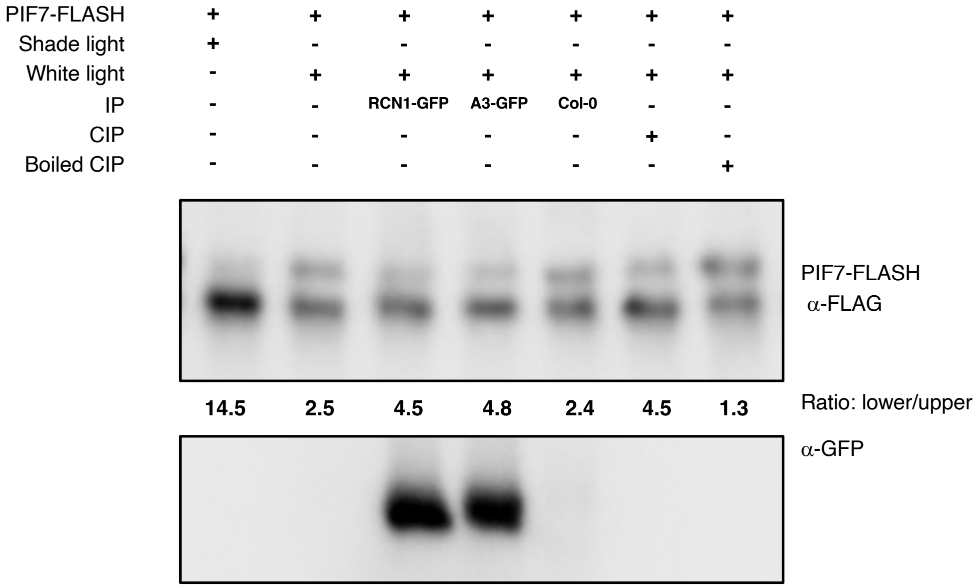

Figure S11: PP2A can dephosphorylate PIF7-FLASH *in vitro.* Dephosphorylation assay was performed using immunoprecipitated PP2A complexes and PIF7-FLASH as substrate. PP2A proteins were immunoprecipitated from *35S:RCN1-GFP*, *35S:A3-GFP* overexpression lines and incubated with PIF7-FLASH-containing extracts from white-light–grown seedlings for 1 h at 30° C. CIP served as a positive control, while boiled CIP and Col-0 IP products served as negative controls. α-FLAG antibody was used for detection. CIP, calf intestinal phosphatase; WL, white light; SL, shade light.

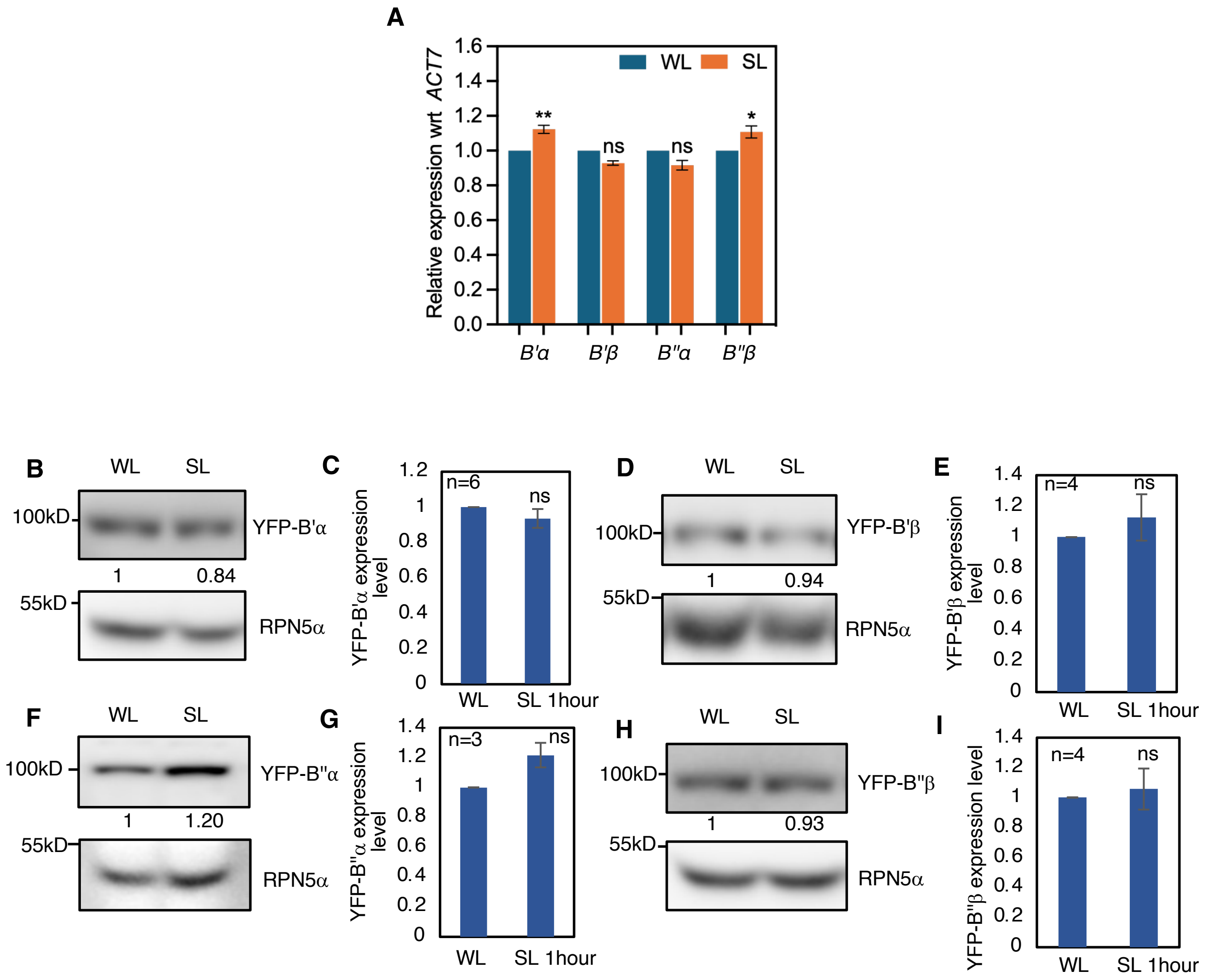

Figure S12: Gene Expression level and protein accumulation of PP2A subunits under shade light condition. (A) RT-qPCR analysis showing transcript levels of *PP2A B′α, B′β, B″α* and *B″β* under white light and SL (1 h) conditions. Seedlings were grown for 4 days under white light then either kept in white light or treated with shade light for 1 hour. Expression levels were normalized to *ACT7*, and relative expression in white light grown seedlings was set to 1. Data represent mean ± SEM (n = 3 biological replicates). A one-way ANOVA followed by Tukey's multiple comparison test was performed. Statistically significant differences are indicated ($P < 0.05$). WL, white light; SL, shade light. **(B-I)** Immunoblots and bar graphs showing the protein expression of YFP-B'α **(B,C)**, YFP-B'β **(D,E)**, YFP-B″α **(F,G)**, and YFP-B″β **(H,I)** under white light and shade light. Four-day-old white-light grown seedlings were either kept in white light or exposed to shade light for 1 h before being sampled for protein extraction. A RPN5α blot was used as the loading control. The numbers indicate the abundance of YFP-B'α, YFP-B'β, YFP-B''α, and YFP-B''β proteins after normalization. WL, white light; SL, shade light. Data represent mean ± SEM. Student's t-test was performed; $P < 0.05$ was considered significant.

Table S1: Primers used in this study

QPCR primers		
ACT7-qPCR-F	CACCGCTCTTGCACCTAG	ACTIN7,AT5G09810, as reference gene
ACT7-qPCR-R	GACCTGACTCATCGTACTCAC	
B''α-qPCR-F	TCCTGAAACTGGCCGATTG	B''α AT5G44090
B''α-qPCR-R	AGTGCCGCTGTTCAGAAA	
B''β-qPCR-F	GTTTGTCTCCTTCTCCCCTC	B''β AT1G03960
B''β-qPCR-R	GGTGTTCCAGATTTAGCATCATC	
B'α-qPCR-F	AGTTCCAACGCTGTATGGTC	B'α AT5G03470
B'α-qPCR-R	GTTCTGAGCTATTAGACCCACC	
B'β-qPCR-F	AATTGGTCGCTGTCTCACTAG	B'β AT3G09880
B'β-qPCR-R	GGATCACGCTTCGGTTTTG	
YUC8-qPCR-F	TGTATGCGGTTGGGTTTACGAGGA	YUC8 AT4G28720
YUC8-qPCR-R	CCTTGAGCGTTTCGTGGGTTGTTT	
YUC9-qPCR-F	CCTCAGACGGAGAGGAGATGGAA	YUC9 AT1G04180
YUC9-qPCR-R	CCCCATAATCTCCCTCGGTAAAACA	
IAA19-qPCR-F	GGTGACAACTGCGAATACGTTACCA	IAA19 AT3G15540
IAA19-qPCR-R	CCGGTAGCATCCGATCTTTTCA	
IAA29-qPCR-F	AGATACAAATTATACATTCACCTTCC	IAA29 AT4G32280
IAA29-qPCR-R	AAACATCTTGTATATGCACACG	
